# *PIF*-independent regulation of growth by an evening complex in the liverwort *Marchantia polymorpha*

**DOI:** 10.1371/journal.pone.0269984

**Published:** 2022-06-16

**Authors:** Ulf Lagercrantz, Anja Billhardt, Sabine N. Rousku, Katarina Landberg, Mattias Thelander, D. Magnus Eklund

**Affiliations:** 1 Plant Ecology and Evolution, Department of Ecology and Genetics, Evolutionary Biology Centre, Uppsala University, The Linnean Centre for Plant Biology in Uppsala, Uppsala, Sweden; 2 Department of Plant Biology, Swedish University of Agricultural Sciences, The Linnean Centre of Plant Biology in Uppsala, Uppsala, Sweden; 3 Physiological Botany, Department of Organismal Biology, Uppsala University, The Linnean Centre for Plant Biology in Uppsala, Uppsala, Sweden; Ohio State University, UNITED STATES

## Abstract

Previous studies in the liverwort *Marchantia polymorpha* have shown that the putative evening complex (EC) genes *LUX ARRHYTHMO* (*LUX*) and *ELF4-LIKE* (*EFL*) have a function in the liverwort circadian clock. Here, we studied the growth phenotypes of Mp*LUX* and Mp*EFL* loss-of-function mutants, to establish if *PHYTOCHROME-INTERACTING FACTOR* (*PIF*) and auxin act downstream of the *M*. *polymorpha* EC in a growth-related pathway similar to the one described for the flowering plant Arabidopsis. We examined growth rates and cell properties of loss-of-function mutants, analyzed protein-protein interactions and performed gene expression studies using reporter genes. Obtained data indicate that an EC can form in *M*. *polymorpha* and that this EC regulates growth of the thallus. Altered auxin levels in Mp*lux* mutants could explain some of the phenotypes related to an increased thallus surface area. However, because Mp*PIF* is not regulated by the EC, and because Mp*pif* mutants do not show reduced growth, the growth phenotype of EC-mutants is likely not mediated via Mp*PIF*. In Arabidopsis, the circadian clock regulates elongation growth via *PIF* and auxin, but this is likely not an evolutionarily conserved growth mechanism in land plants. Previous inventories of orthologs to Arabidopsis clock genes in various plant lineages showed that there is high levels of structural differences between clocks of different plant lineages. Here, we conclude that there is also variation in the output pathways used by the different plant clocks to control growth and development.

## Introduction

Circadian clocks initiate biological rhythms to enable anticipation of cycles of light and temperature, and to time biological processes. The circadian clock is a self-sustaining oscillator and the approximately 24-hour rhythm results mainly from a network of transcriptional and translational feedback loops [1]. In flowering plants, one key component of this network is the evening complex (EC). The EC in *Arabidopsis thaliana* has been described to consist of three proteins: EARLY FLOWERING 3 (ELF3), ELF4, and LUX ARRHYTHMO (LUX) [2–4]. This complex has a vital role in the circadian clock, and, unlike other clock genes in Arabidopsis, knockout mutants of these three genes show an arrhythmic phenotype. The EC has been shown to regulate the circadian clock by repressing the core clock genes *TIMING OF CAB EXPRESSION 1* (*TOC1*), *PSEUDO-RESPONSE REGULATOR7* (*PRR7*), *PRR9*, and *LUX* during the night [5–7]. As the morning components CIRCADIAN CLOCK ASSOCIATED 1 (CCA1)/LATE ELONGATED HYPOCOTYL (LHY) represses the EC, a transcriptional feedback loop is formed [4, 5, 8–10]. The EC also have functions outside the circadian clock, as mutants of EC genes also show alterations in cell expansion and early flowering [3, 11–13].

An important function of the circadian clock and the EC is in the control of elongation growth of seedlings [12–14]. The EC represses *PHYTOCHROME INTERACTING FACTOR 4* (*PIF4*), which is a key transcription factor promoting growth in Arabidopsis [12, 13]. PIF4 regulates growth through auxin biosynthesis and signaling [15, 16], as well as through brassinosteroids (BR) and gibberellic acids (GA) [17–19]. Light plays an important role in PIF function as light-activated phytochrome B (phyB) phosphorylates PIF4, resulting in the degradation of PIF4 via the 26S proteasome pathway [20]. As the EC shows circadian rhythm with a peak in the evening, and PIF4 is degraded in light, cell elongation is promoted mainly at late night [12, 13]. The regulation of growth by the EC through *PIF4* is also reported to be temperature dependent, as high temperatures reduce the repression of *PIF4* by attenuated binding of EC to the *PIF4* promoter [13, 21].

In Arabidopsis, circadian clocks are to some extent tissue specific, so that the clock in vascular tissue is most important for regulation of photoperiodic flowering, while the epidermal clock is more important in the control of cell elongation [22, 23]. Endo et al. [22] further suggested that the EC resides primarily in vasculature. This conclusion is supported by the high and circadian expression of *ELF4* only in vasculature [22], and that *LUX* shows a peak of expression at dusk only in vascular tissue [23]. In the mesophyll, *LUX* expression is shifted to the morning as in *cca1*/*lhy* double mutants [23, 24]. Thus, the repression of *LUX* by CCA1 might be confined to vasculature tissue in Arabidopsis. In contrast, it has been reported that the temperature dependent growth control mediated through *PIF4* is active in the epidermis [25]. If this control involves parts of the EC, or the complete EC, is still unclear, but some evidence for a role of epidermal ELF3 have been reported [25].

We have recently shown that single *ELF3*, *ELF4* and *LUX* homologs, exhibiting the domains typical for their respective gene families, exist in the genome of the liverwort *Marchantia polymorpha*, and that such homologs are present in all major plant taxa since the origin of charophytes, with the exception of *ELF3* homologs which have not been found in the gymnosperm clade [26]. As these genes likely have one major function as a complex in vascular tissue and another function in epidermal tissue of Arabidopsis [22, 25], it is of specific interest to characterize basic EC functions in a species that lacks vascular tissue. Among the non-vascular land plants, the liverwort *Marchantia polymorpha* has recently emerged as a model species with low genetic redundancy among regulatory genes, simplifying genetic approaches [27]. Many molecular tools are available for genome editing and loss- and gain-of-function analyses [28–31], as well as tools for studying growth and clock output [32, 33].

We previously confirmed that the EC genes have a function in the *M*. *polymorpha* circadian clock by generating knock-down lines of Mp*LUX* and Mp*ELF4-LIKE* (Mp*EFL*) using artificial micro-RNA’s (amiR’s) [34, 35], as well as knock-out lines of Mp*LUX* using CRISPR/Cas9 [30, 32]. However, outside flowering plants, a role for EC genes in the control of growth has not been established.

In the present study, we conclude that a protein complex comprising MpELF3, MpEFL and MpLUX likely can form. We further show that Mp*EFL* and Mp*LUX* have important functions in the control of thallus growth. However, Mp*PIF* is reported not to affect growth in *M*. *polymorpha* [36], and in accordance we found no evidence for Mp*PIF* acting downstream of the EC to regulate hormonal and other responses, leading to growth of the liverwort thallus.

## Results

### *LUX ARRHYTHMO* and *ELF4-LIKE* regulate growth rates of the gametophyte in *Marchantia polymorpha*

**Fig 1:**
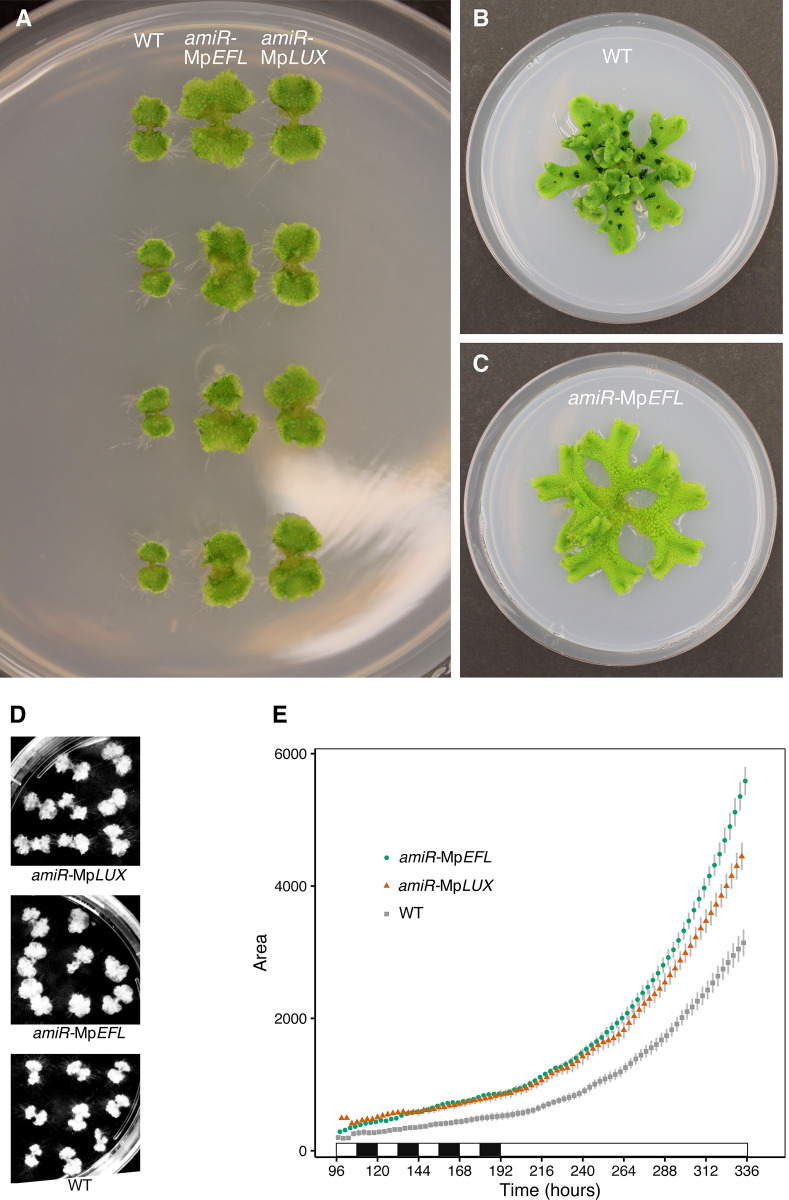
Evening complex knock-down mutants *EF1*_*pro*_:*amiR-*Mp*EFL*^MpMIR160^ and *EF1*_*pro*_:*amiR-*Mp*LUX*^MpMIR160^ display increased growth rate in *Marchantia polymorpha* gemmalings. (A) 2-week-old gemmalings of wild type (Upp5), *EF1*_*pro*_:*amiR-*Mp*EFL*^Mp*MIR160*^ and *EF1*_*pro*_:*amiR-*Mp*LUX* ^Mp*MIR160*^ grown in ND on standard growth medium. (B) 6-week-old wild-type (Upp14) plant grown in LD on standard growth medium. (C) 6-week-old *EF1*_*pro*_:*amiR-*Mp*EFL* ^Mp*MIR160*^ *#2* plant grown in LD on standard growth medium. (D) Images of assayed plants at end of growth-rate experiment shown in (E). (E) Mean ± SE of gemmaling area plotted against time after plating for wild type, *EF1*_*pro*_:*amiR-*Mp*EFL* ^Mp*MIR160*^ and *EF1*_*pro*_:*amiR-*Mp*LUX* ^Mp*MIR160*^ gemmalings. Graphs are based on the gemmalings shown in (D). Statistical analysis revealed a highly significant difference in slope; *P* < 2x10^-16^ between both knock-down lines and wild type. Plants were grown in ND for 8 days and then LL for an additional 6 days. Petri dishes are 9 cm wide.

**Fig 2:**
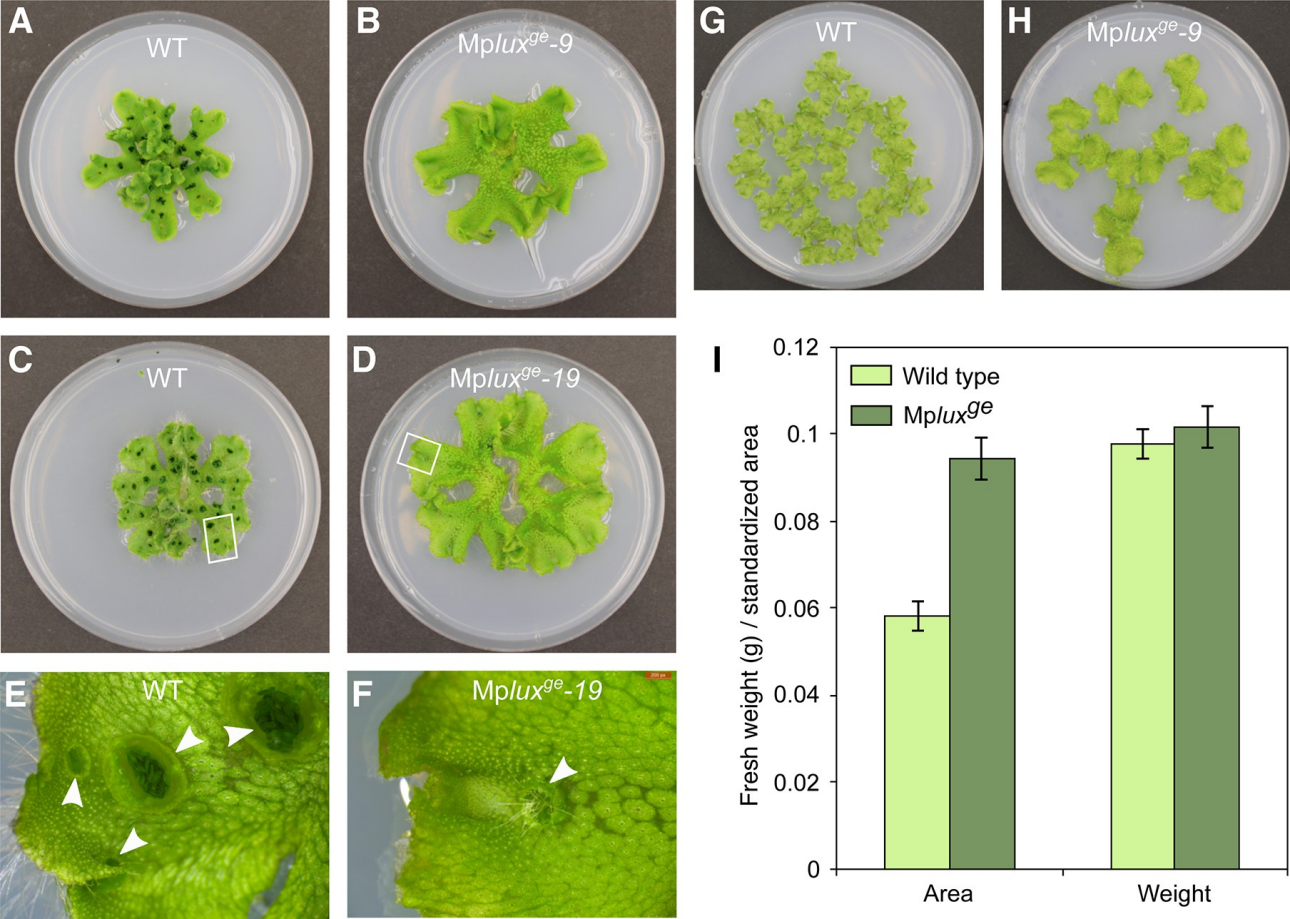
*Marchantia polymorpha lux*^*ge*^ knock-out mutants show increased growth, reduced gemma cup formation and reduced gemma dormancy. (A) 6-week-old wild-type plant grown in LD on standard growth medium (same plant as in Fig 1B). (B) 6-week-old Mp*lux*^*ge*^*-9* plant grown in LD on standard growth medium. (C) 6-week-old wild-type (Upp14) plant grown in LD on medium supplemented with 2% sucrose. (D) 6-week-old Mpl*ux*^*ge*^*-19* plant grown in LD on medium supplemented with 2% sucrose. (E) Close-up of boxed area in (C), showing gemma cups with dormant gemmae. (F) Close-up of boxed area in (D), showing a single gemma cup with protruding rhizoids indicating germinating gemmae. (G,H) 3-week-old gemmalings grown in LD on standard growth medium, used to produce data shown in (I). For these measurements we used Upp13 (G) and 14, and Mp*lux*^*ge*^*-9* (H) and *19*. (I) Measurements of area and weight of wild type and Mp*lux*^*ge*^. Mean area and weight (n = 16 for Upp; n = 14 for Mp*lux*^*ge*^) is given ± SE. Two-tailed t-test for area and weight *P* = 4 x 10^−11^ and *P* = 0.5, respectively. Petri dishes are 9 cm wide.

To learn more about how Mp*EFL* and Mp*LUX* affect growth of the gametophyte thallus we grew gemmalings from several independent lines of *EF1*_*pro*_:*amiR-*Mp*LUX*^Mp*MIR160*^ and *EF1*_*pro*_:*amiR-*Mp*EFL*^Mp*MIR160*^ axenically on petri dishes containing standard growth medium. After 14 days in neutral day photoperiod (ND; 12:12 h light:darkness) we observed an increased surface area of the thallus in all amiR-lines compared to wild type (Fig 1A). The enlarged thallus was maintained throughout the adult life of the loss-of-function lines, and was also observed after 6 weeks of growth in long day photoperiod (LD) (Fig 1B and 1C). Growth rate analysis, measuring the observable thallus area of gemmalings from wild type and the amiR-lines (Fig 1D), showed that the highly efficient *EF1*_*pro*_:*amiR-*Mp*EFL*^Mp*MIR160*^ construct, reducing Mp*EFL* mRNA levels with >90% [35], resulted in growth rates significantly higher than both *EF1*_*pro*_:*amiR-*Mp*LUX*^Mp*MIR160*^ and wild type (*P* < 2x10^-16^; Fig 1E). Plants harboring the less efficient *EF1*_*pro*_:*amiR-*Mp*LUX*^Mp*MIR160*^ construct (>60% lower mRNA levels compared to wild type) [35] displayed growth rates significantly higher than wild type but lower than *EF1*_*pro*_:*amiR-*Mp*EFL*^Mp*MIR160*^ (*P* < 2x10^-16^; Fig 1E). We therefore included the Mp*LUX* knock-out mutants Mp*lux*^*ge*^*-9* and Mp*lux*^*ge*^*-19* in the study. These were identical to each other, and very similar to that of *EF1*_*pro*_:*amiR-*Mp*EFL*^Mp*MIR160*^ lines (Figs 1B, 1C, 2A and 2B). Hence, the Mp*LUX* knock-out mutants showed a similar but more severe phenotype than the Mp*LUX* knock-down lines.

In adult Mp*lux*^*ge*^ and amiR-Mp*EFL* lines we observed epinastic curvature of the apical region of thalli as well as along the midrib, similar to auxin overproducing lines [37]. The thalli of the loss-of-function-lines appeared to have a lighter green color than the wild type, and the plants did not produce gemma cups when grown on standard growth medium (Figs 1B, 1C, 2A and 2B). However, cups were occasionally produced in Mp*lux*^*ge*^ after the second bifurcation event, when grown on medium supplemented with sucrose (Fig 2C–2F). Gemma cups in the loss-of-function lines were initially smaller than wild-type cups, and rhizoids protruding out of all observed mutant cups (n > 15) suggested that gemmae germinate inside the cups immediately after maturation (Fig 2E and 2F). Wild-type cups of similar age or size never contain protruding rhizoids when grown in identical growth conditions as the Mp*lux*^*ge*^ mutants. Hence, dormancy is likely not established in Mp*lux*^*ge*^ gemmae, allowing light to initiate germination of newly produced gemmae inside the cup [38]. Alternatively, the light-induced germination signal is already present in the newly produced gemmae as they reach maturity in cups of the Mp*lux*^*ge*^ mutant. Because *EF1*_*pro*_:*amiR-*Mp*EFL*^Mp*MIR160*^ and Mp*lux*^*ge*^ thalli appeared less rigid than wild-type thalli we measured the weight and area of 3-week-old wild-type and Mp*lux*^*ge*^ gemmalings. We found that the average weight of the mutant gemmalings was similar to wild type. Because Mp*lux*^*ge*^ gemmalings have a larger area than wild type due to an increased growth rate, we observed a relative weight to area ratio of 0.65 in Mp*lux*^*ge*^ as compared to wild type (Fig 2G–2I).

### *Marchantia polymorpha LUX* loss-of-function lines have a wider and thinner thallus than wild type and have a reduced number of chlorenchyma cells in air chambers

**Fig 3:**
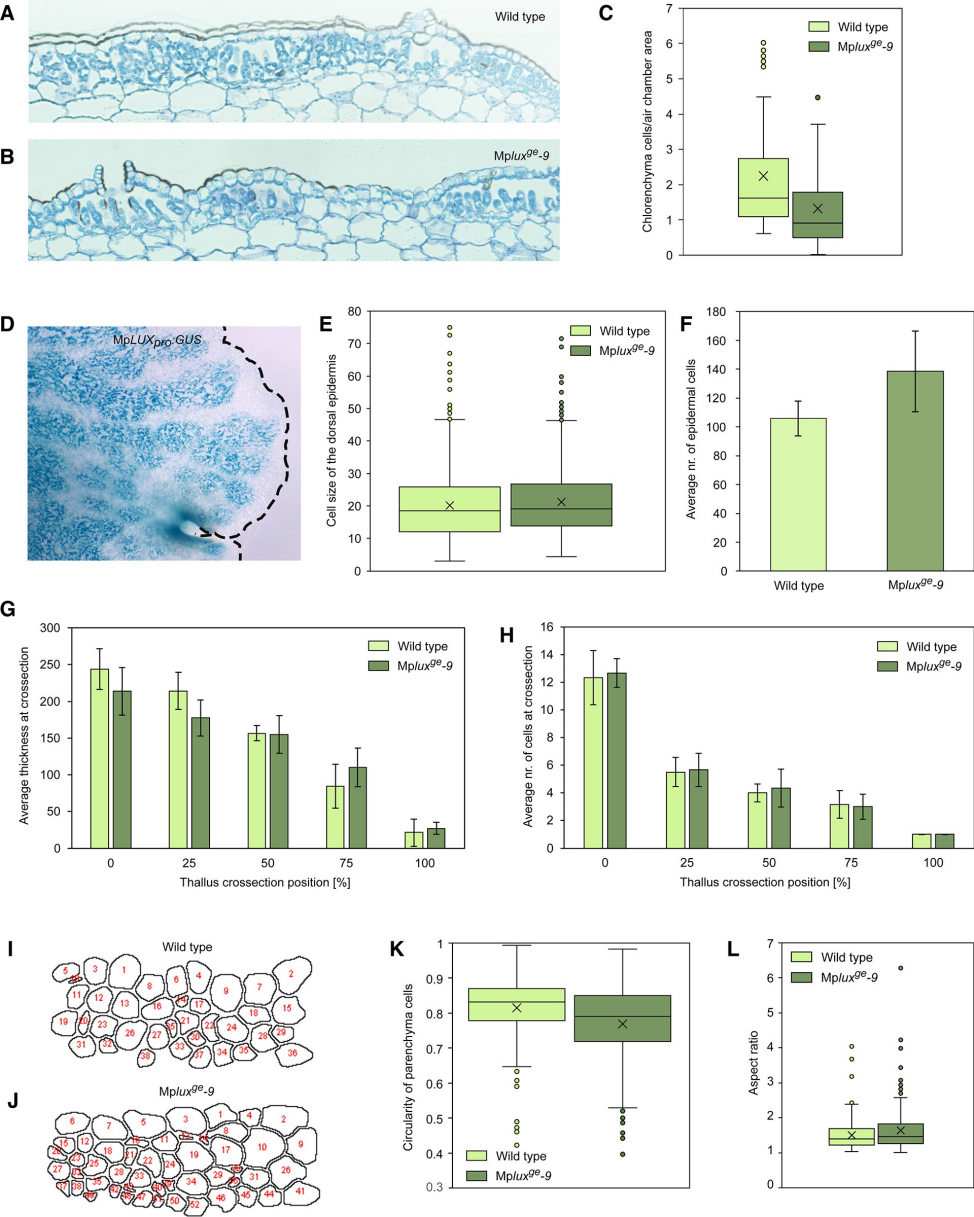
Differences in cell number, size and structure explain the growth phenotype of the *Marchantia polymorpha lux*^*ge*^ knock-out mutant. (A,B) Micrographs of representative transverse sections displaying the number and size of dorsal epidermis, air pores, air chamber area and the underlying parenchyma in the wild type (A), and in Mp*lux*^*ge*^*-9* (B). (C) Boxplot of chlorenchyma cells per air chamber area unit in wild type (number of analyzed air chambers, n = 60) and Mp*lux*^*ge*^*-9* (n = 38). Two-tailed t-test, *P* = 0.0019. (D) Apical region of GUS-stained 3-week-old thallus from Mp*LUX*_*pro*_:*GUS#7*. Dashed line indicates the thallus margin. (E) Boxplot showing the average dorsal epidermal cell width in wild type (number of analyzed epidermal cells, n = 1058) and Mp*lux*^*ge*^*-9* (n = 831). Two-tailed t-test, *P* = 0.028. (F) Graph showing the average number of epidermal cells in transverse sections of wild type (number of analyzed thalli, n = 10) and Mp*lux*^*ge*^*-9* (n = 6). Two-tailed t-test, *P* = 0.0054. (G) Graph of average relative thickness of the thallus at 0 (midrib), 25, 50, 75 and 100% of the thallus length in wild type (number of analyzed thalli, n = 6) and Mp*lux*^*ge*^*-9* (n = 7). Two-tailed t-test, *P*(25) = 0.029. (H) Graph of the average number of cells at the five positions indicated in (G). (I,J) Pictures of parenchyma cell perimeters in a rectangle between 0 and 25% of the total thallus length in the transverse sections analyzed in (G,H). Wild type (I) and Mp*lux*^*ge*^*-9* (J). (K,L) Boxplots showing the average circularity (K) and aspect ratio (L) of cells in wild type (number of analyzed cells, n = 235) and Mp*lux*^*ge*^*-9* (n = 270) shown in (I,J). Two-tailed t-test, *P* < 0.001 (K), *P* = 0.003 (L). Error bars in (F,G,H) shows SD. Bars in the boxplots (D,E,K,L) shows max and min values, with outliers shown as dots. Boxplots show average as x, and median as a line. Boxes reach from the 25^th^ to the 75^th^ percentile.

To investigate the growth phenotype of the Mp*LUX* and Mp*EFL* loss-of-function mutants in more detail we sectioned the thallus of 3-week-old Mp*lux*^*ge*^ plants. Because the two independent mutant lines were identical at the macroscopic level, and clearly different from all male and female wild-type lines available, we decided to analyze only the Mp*lux*^*ge*^*-9* mutant and one wild-type line in more detail. Transverse sections 240 to 480 μm from the apical region revealed a striking difference in the number of chlorenchyma cells in air chambers of the Mp*lux*^*ge*^*-9* mutant compared to the wild type (Fig 3A–3C; S1 Fig). The mutant had an average of 1.3 chlorenchyma cells per air chamber area unit in the transverse sections, compared to 2.2 chlorenchyma cells per area unit in the wild type, corresponding to a 40% decrease (two-tailed t-test, *P* = 0.0019; Fig 3C). There was no difference between the wild type and Mp*lux*^*ge*^*-9* in the area of measured air chambers in these transverse sections (two-tailed t-test, *P* = 0.26; S2 Fig). The reduced number of small chloroplast dense chlorenchyma cells in photosynthetic filaments close to the dorsal epidermis likely explains the lighter green color of the loss-of-function lines compared to the wild type. Because of this striking phenotype, we generated several independent Mp*LUX*_*pro*_:*GUS* lines to test if Mp*LUX* is expressed in the photosynthetic filaments of the air chambers. All analyzed GUS-lines showed an identical and clear spatial expression domain in the filament cells, in developing gemmae and cells in apical notch regions (Fig 3D; S3 Fig).

In addition to color, a striking phenotype of Mp*lux*^*ge*^ was the enlarged surface area of the thalli. When measuring the width, and counting the number of dorsal epidermal cells from the midrib to the tip of the thallus margin in transverse sections, we observed a slight (*c*. 5%), but significant, increase of the average cell width in Mp*lux*^*ge*^ compared to the wild type (two-tailed t-test, *P* = 0.028; Fig 3E). In this epidermal layer we also observed a significantly increased number of cells in Mp*lux*^*ge*^*-9* (two-tailed t-test, *P* = 0.0054; Fig 3F; S4 Fig), suggesting that the mutant thallus area is larger due to both more and wider cells.

The increased surface area in combination with maintained weight prompted us to analyze the dorsiventral thickness of the thallus in the transverse sections. Because air chambers contain two cell layers, with air and filamentous cells in between, making measurements of thickness difficult, we measured the thickness of the thallus below the air chamber at five positions correlating to 0, 25, 50, 75 and 100% of the total distance from the midrib to the tip in the sections (S5 Fig). We observed a slight, but significant, decrease of the average dorsiventral thickness at 25% of the total thallus length in Mp*lux*^*ge*^ (Fig 3G; two-tailed t-test, *P* = 0.029). However, we did not observe a difference in cell number between Mp*lux*^*ge*^ and wild type (Fig 3H), suggesting that the parenchyma cells in the central region of the Mp*lux*^*ge*^ thallus have a different shape and/or size. To examine this we first measured the circularity of parenchyma cells in a rectangle between the 0% (midrib) and 25% positions (S5 Fig). We could determine that parenchyma cells in the wild type were significantly more circular than parenchyma cells in the Mp*lux*^*ge*^ mutant (Fig 3I–3K). We also measured aspect ratio (AR) in the same data set and found that wild type had an average AR of 1.49, while the Mp*lux*^*ge*^ mutant had a significantly higher AR of 1.63 (Fig 3L; two-tailed t-test, *P* = 0.003). The cell shapes in the mutant line thus appear more flattened than in the wild type, which could explain a reduced thickness while maintaining the number of cells.

### *Marchantia polymorpha PIF* is unlikely to mediate the growth effect of the evening complex genes in Mp*lux*^*ge*^ loss-of-function mutants

**Fig 4:**
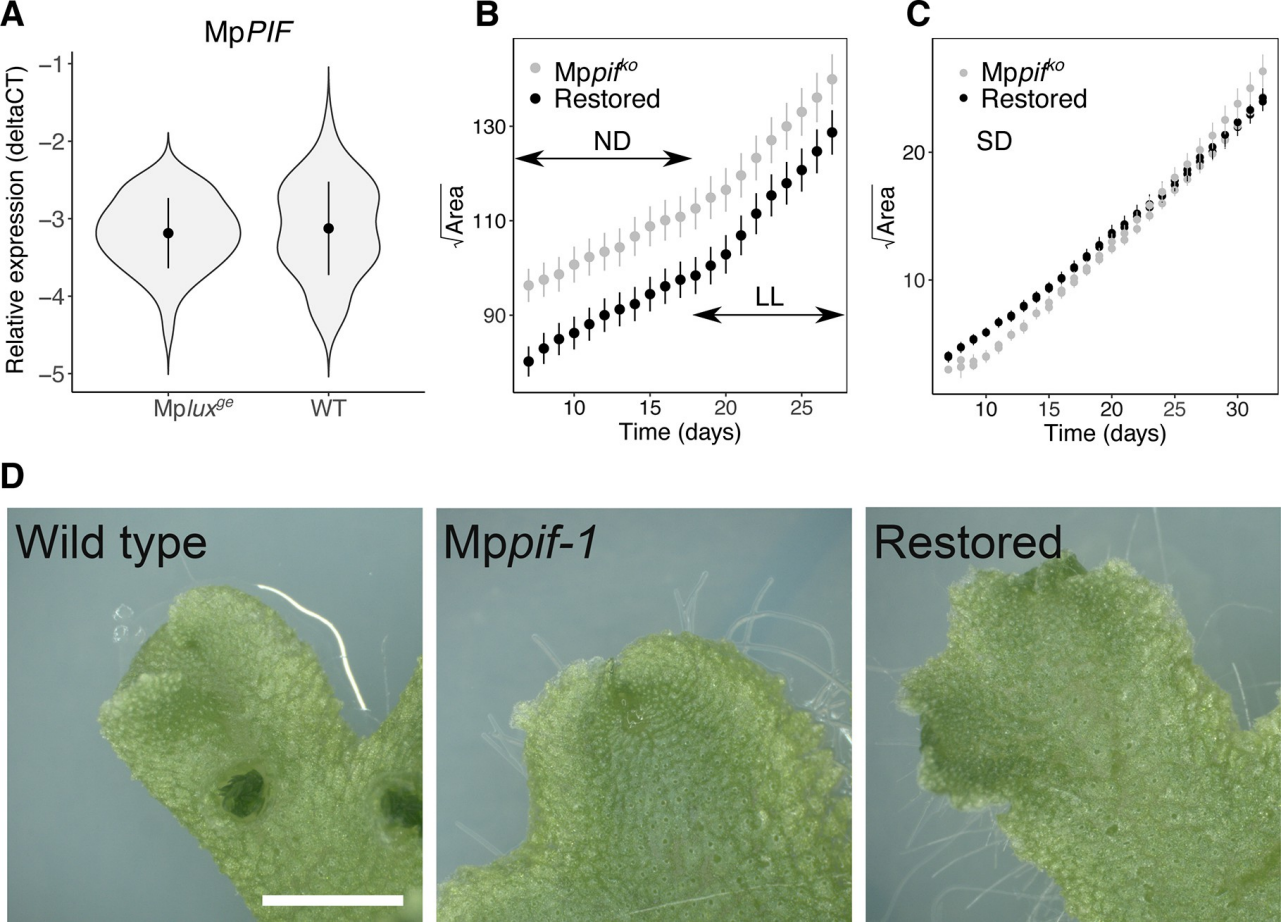
Altered growth of *Marchantia polymorpha lux*^*ge*^ knock-out mutants is not mediated through Mp*PIF*. (A) Graph shows the average expression of Mp*PIF* in wild type (Upp1 and Upp5) and Mp*lux*^*ge*^ mutants *9* and *19*, measured by qRT-PCR. (B) Square root of area plotted over time for Mp*pif*^*ko*^*-1* and restored line Mp*pif*^*ko*^*/*Mp*PIF*_*pro*_:Mp*PIF-1*. Each point is an average ± SD of 11 and 18 gemma for Mp*pif* and restored line, respectively. Statistical analysis revealed no difference in slope (*P* > 0.8). ND, neutral days. LL, continuous light. (C) Square root of area plotted over time for Mp*pif*^*ko*^*-1*, Mp*pif*^*ko*^*-2* and restored lines Mp*pif*^*ko*^*/*Mp*PIF*_*pro*_:Mp*PIF-1*, Mp*pif*^*ko*^*/*Mp*PIF*_*pro*_:Mp*PIF-2*. Statistical analysis gave a *P* < 10^−15^ for equal slopes. SD, short days. Each point is an average ± SD of 12, 12, 11, 7 gemma for Mp*pif* and restored lines, respectively. (D) Apical regions of adult wild type (Tak-1), Mp*pif*^*ko*^*-1* and restored line Mp*pif*^*ko*^*/*Mp*PIF*_*pro*_:Mp*PIF-2*. Bar 0.5 cm.

In Arabidopsis, PIF4 and PIF5 promotes elongation growth of hypocotyls, and both genes act downstream of light- and clock-signaling pathways [16, 39]. Specifically, repression of *PIFs* is mediated by the EC. To test if Mp*PIF* act downstream of the EC in *M*. *polymorpha* we first used qRT-PCR to analyze the average expression levels of Mp*PIF* in gemmalings of two wild type accessions and two Mp*lux*^*ge*^ mutants entrained in neutral day photoperiod (ND) and sampled over two days in constant light (LL). We found no difference between the mutants and wild types (Fig 4A). Secondly, we analyzed the growth rate of an Mp*pif*^*ko*^ mutant compared to a restored line Mp*PIF*_*pro*_:Mp*PIF* Mp*pif*^*ko*^, described to behave as the wild type [38]. Although the projected area of the Mp*pif*^*ko*^ mutant was larger at the start of the imaging in ND, we found no difference in growth rate in ND and LL (Fig 4B; statistical analysis revealed no difference in slope; *P* > 0.8), suggesting that Mp*PIF* is not controlling growth rate of the thallus in *M*. *polymorpha* under these conditions. As MpPIF, similarly to PIF proteins in Arabidopsis, is degraded in light [38], we repeated the experiment in short day (SD) conditions with recently germinated gemmae, to maximize a potential effect of MpPIF on growth (Fig 4C). We have previously shown that *M*. *polymorpha* gemmalings are not expanding in constant darkness and therefor this growth condition was not tested [35]. Under SD conditions we found a limited, but significantly, higher growth rate in two independent Mp*pif*^*ko*^ mutant as compared to two restored lines (statistical analysis gave a *P* < 10^−15^ for equal slopes). Importantly, these two restored lines show identical growth and response to light signals as the wild type [38]. Recently, Hernandez-Garcia et al. [36] suggested that the control of *M*. *polymorpha* thallus size is largely independent of Mp*PIF*. Taken together, these results suggest the enlarged growth phenotype of Mp*lux*^*ge*^ and *EF1*_*pro*_:*amiR-*Mp*EFL*^Mp*MIR160*^ is not mediated via the single Mp*PIF* gene. Additionally, we did not observe a difference in color between the Mp*pif* knock-outs, the restored lines and wild type, suggesting chlorenchyma filaments in air chambers are not affected in these transgenic lines (Fig 4D).

### *Marchantia polymorpha lux*^*ge*^ loss-of-function mutants show auxin-related phenotypes

**Fig 5:**
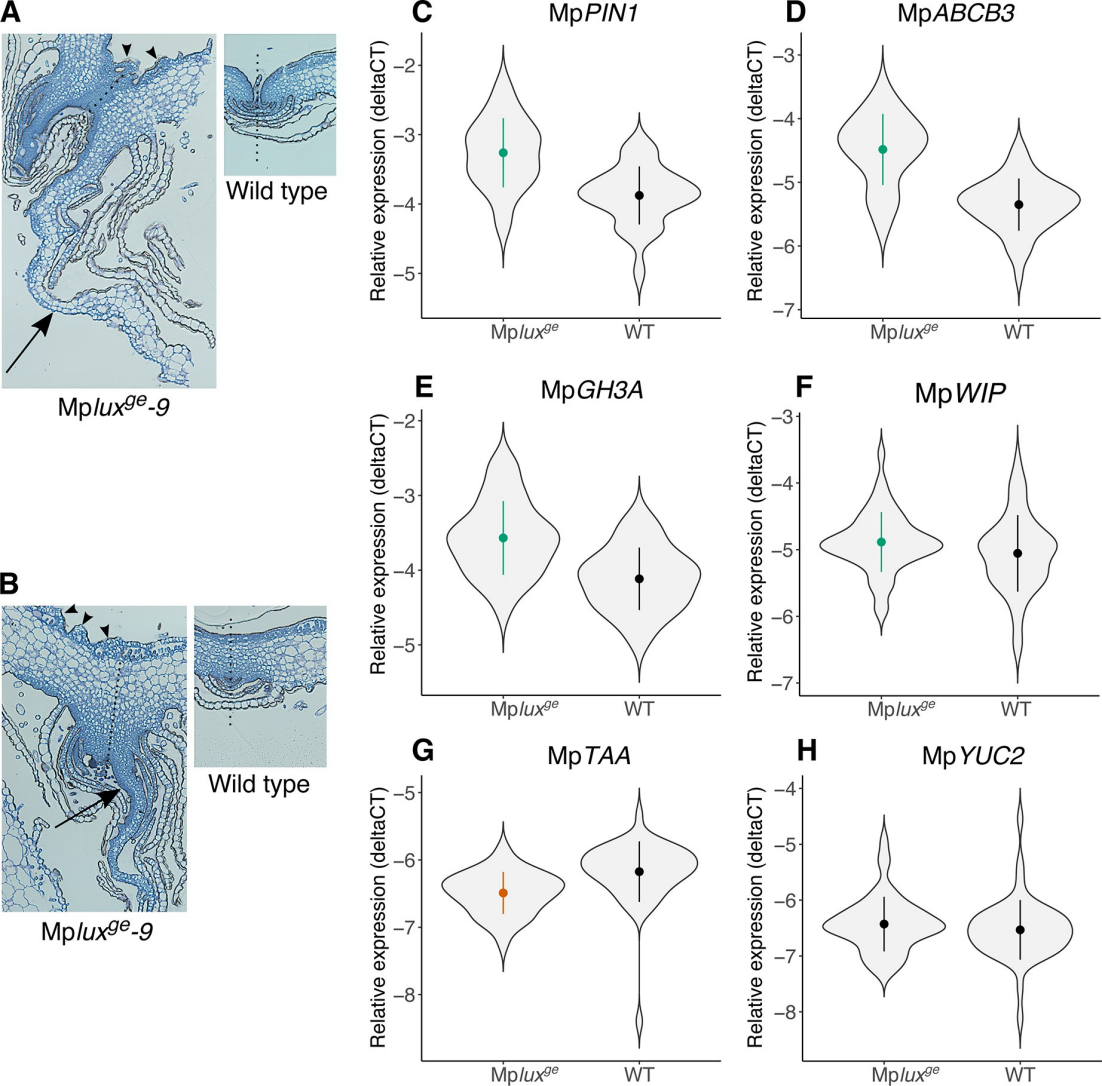
*Marchantia polymorpha lux*^*ge*^ knock-out mutants show auxin related phenotypes and altered auxin responses. (A,B) Micrographs of representative transvers sections of Mp*lux*^*ge*^*-9* and wild type, close to the bottom of the apical notch (A), and about 120 μm behind the apical notch (B). Similar growth patterns were observed in several biological replicates. Dotted lines denote the midrib. Arrows point at aberrant outgrowth of lobe-like tissue where ventral scales are expected. Arrowheads point at protrusions of the epidermal layer. (C-H), Gene expression in wild type (Upp1 and Upp5) and Mp*lux*^*ge*^*-9* and *-19* measured by qRT-PCR. (C) Mp*PIN1*. (D) Mp*ABCB3*. (E) Mp*GH3A*. (F) Mp*WIP*. (G) Mp*TAA*. (H) Mp*YUC2*. Average expression was estimated from normalized dCT values over 12 time points in a 44 hour time series (n = 4). Green and red symbols indicate significantly higher and lower expression as compared to wild type, respectively. *P*-values for genotype effect in ANOVA were: 8.9x10^-15^ (Mp*PIN1*); 2.1x10^-14^ (Mp*ABCB3*); 1.4x10^-9^ (Mp*GH3A*); 0.04 (Mp*WIP*); 5.5x10^-5^ (Mp*TAA*); 0.24 (Mp*YUC2*).

It is well established that auxin affects growth in plants (see for example the reviews [40, 41], and references therein), and we have previously shown that auxin levels in wild-type *M*. *polymorpha* gemmalings show a clear circadian rhythm in LL conditions [32]. However, in Mp*lux*^*ge*^ lines, auxin levels are higher, arrhythmic and increasing with time in LL [32]. This shows that clock components affect production and/or inactivation of IAA. Inspection of several independent transversal sections of Mp*lux*^*ge*^ close to the apical notch revealed bulging and protrusion of newly developing air chambers, possibly resulting from exaggerated enlargement of the epidermal cell layer (Fig 5A and 5B). This observation is consistent with the measurements of epidermal cells that were both slightly larger and more numerous in the Mpl*ux*^*ge*^ mutant than in wild type (Fig 3). Protrusion of air chambers and gemma cups has previously been attributed to increased auxin levels and signaling [37, 42, 43]. Also, gemmae supplemented with exogenous auxin during germination shows increased rhizoid production [42], and gemmae kept in darkness will germinate if placed on an auxin source [44]. This suggests that premature germination of gemmae in cups of the Mpl*ux*^*ge*^ mutant could be the result of elevated auxin signalling in developing and/or newly matured gemmae. However, gemmae cups did not display the elongation typically seen in plants with increased auxin signalling (Fig 2F) [42].

The Mpl*ux*^*ge*^ sections also showed conspicuous outgrowth of lobe-like structures where ventral scales are normally found (Fig 5A, 5B). These extra lobe-like structures likely contribute to the overall increased thallus size of Mpl*ux*^*ge*^ plants, and might also be involved in the apparent downward bending of thallus margins that macroscopically could be interpreted as epinastic growth, which is typical for thalli experiencing enhanced levels of auxin [42].

To test if the growth phenotype of the Mp*lux*^*ge*^ lines was also reflected in a disruption of normal auxin signaling, we analyzed expression levels for several auxin related genes in LL using qRT-PCR. In accordance with increasing auxin levels in Mp*lux*^*ge*^ as compared to the wild type, we detected increased expression levels of auxin transport, homeostasis and signaling genes (Mp*PIN1*, Mp*ABCB3*, Mp*GH3A*, Mp*WIP*; Fig 5C–5F). When analyzing the two auxin synthesis genes Mp*YUC2* and Mp*TAA*, no difference in expression was detected for Mp*YUC2*, but expression of Mp*TAA* was significantly reduced in Mp*lux*^*ge*^, which might be explained by feedback regulation due to the rising auxin levels (Fig 5G and 5H) [32].

### An evening complex in *Marchantia polymorpha*

**Fig 6:**
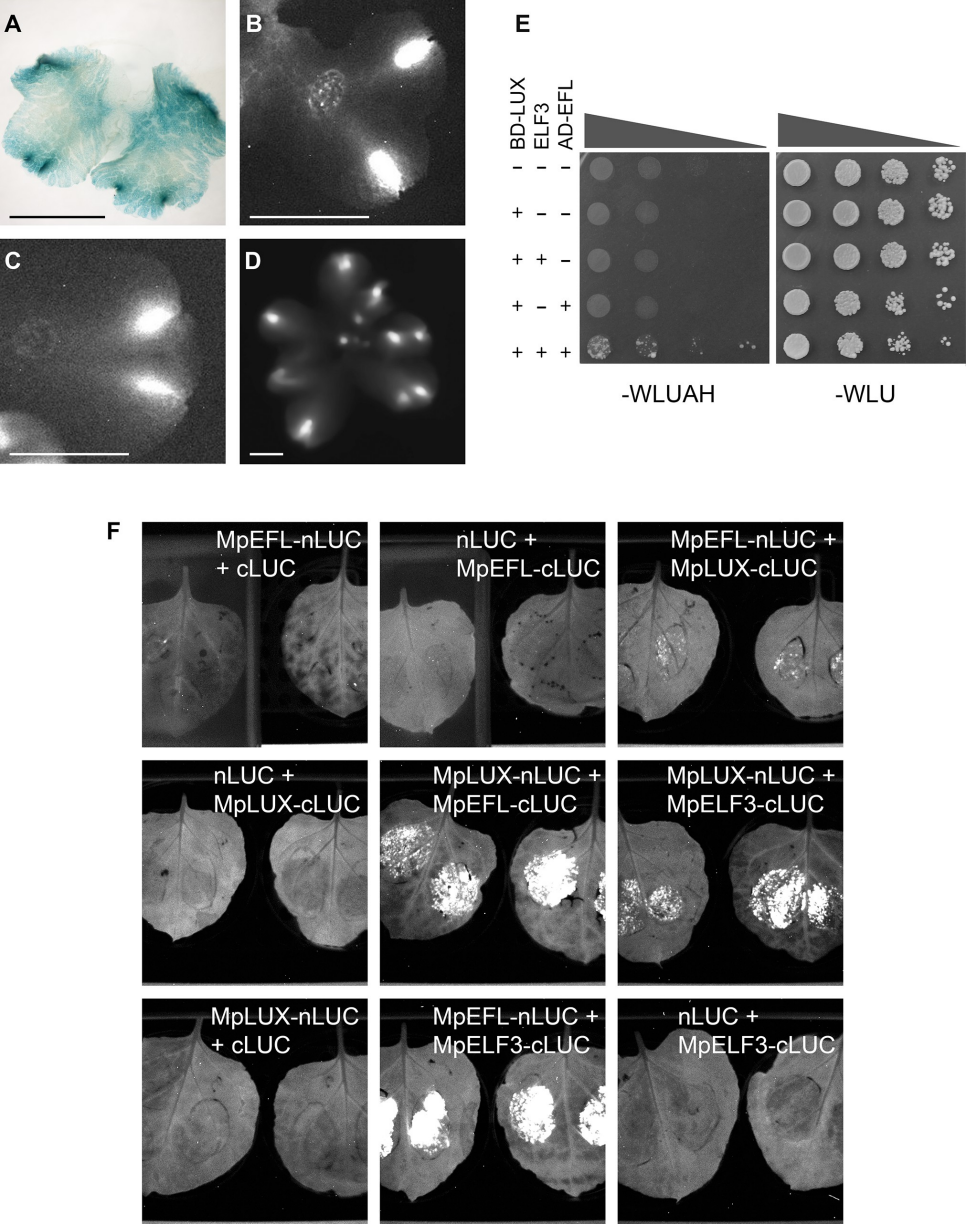
Overlap of expression domains and physical interaction between EC members MpLUX, MpELF3 and MpEFL. (A) Mp*LUX*_*pro*_:*GUS#9*. (B) Mp*LUX*_*pro*_:*LUC*. (C) Mp*ELF3*_*pro*_:*LUC*. (D) Mp*EFL*_*pro*_:*LUC*. (E) The three EC homologs from *M*. *polymorpha* are able to form a complex in a yeast three-hybrid assay.–WLU, selection for combinations of empty plasmids and plasmids expressing the three EC members, as indicated to the left.–WLUAH, selection for interaction between the BD and AD containing proteins using markers *HIS3* and *ADE2*. Transformed yeast were plated in a dilution series and incubated in 30°C for three days (-WLU), or five days (-WLUAH). The AD-EFL construct appears to reduce yeast growth. (F) Split-LUC assays in tobacco leaves displaying plasmid combinations as indicated in the figure. White signal is bioluminescence from reconstituted LUC. Bars in (A-D) are 0.5 cm.

Because loss-of-function lines for the putative EC members MpLUX and MpEFL display identical phenotypes it is plausible that they, possibly together with MpELF3, participate in forming a protein complex in *M*. *polymorpha*. Mp*LUX* and Mp*ELF3* were previously shown to have spatially overlapping expression patterns in the apical region of young gemmalings, in addition to their similar temporal expression patterns [26]. Mp*LUX*_*pro*_:*GUS* transformants revealed that the Mp*LUX* promoter has a weak activity in all cell types of the thallus (S3 Fig). However, Mp*LUX*_*pro*_:*GUS* signal appeared stronger in the apical thallus, in and around the apical notch, but also in photosynthesizing filaments in air chambers further away from the notch and in developing gemmae (Figs 3D and 6A; S3 Fig). To verify overlapping spatial expression domains between all three putative EC-members, we produced Mp*EFL*_*pro*_:*LUC* transformants that confirmed highly overlapping spatial domains between all three genes, both in and around the apical notch, and also weaker signals more broadly in the thallus (Fig 6A–6D) [26]. This indicates that the protein products of Mp*EFL*, Mp*LUX* and Mp*ELF3* are present in the same spatiotemporal domain in the apical thallus, and possibly also in older parts of the thallus.

We subsequently performed protein interaction studies of all three proteins in the yeast *Saccharomyces cerevisiae*. In yeast, MpLUX, fused N-terminally to the GAL4 DNA-binding domain, and MpEFL, fused N-terminally to the GAL4 activation domain, could only interact with each other in the presence of MpELF3 (Fig 6E). We also tested that LUX, single or in the presence of ELF3, did not auto activate transcription of the two selection genes. The yeast 3-hybrid thus shows that MpLUX, MpELF3 and MpEFL can form a complex when expressed in yeast and that the interaction between MpEFL and MpLUX requires MpELF3, as previously suggested for the Arabidopsis EC [13].

Next, we used a split-LUC approach to assess pairwise EC interactions *in planta* [45]. Tobacco (*Nicotiana benthamiana*) leaves were infiltrated with Agrobacterium harboring plasmids expressing MpLUX, MpEFL or MpELF3 C-terminally fused to either the N- or C-terminal half of LUC. Signals from reconstituted LUC were detected for interactions between MpLUX-MpELF3 and MpELF3-MpEFL, but also for MpLUX-MpEFL (Fig 6F). None of the negative controls showed any LUC signal, but all leaves showed EGFP and eqFP611 signals at the infiltration site, originating from marker genes in the backbones of the infiltrated plasmids (S6 Fig). Because the MpLUX-MpEFL interaction was not detected in yeast, unless MpELF3 was co-expressed, the split-LUC signal could be the result of an indirect interaction between MpLUX and MpEFL, bridged by a protein not normally found in yeast, such as *N*. *benthamiana* ELF3.

Even though our experiments are not conclusive on how these three proteins physically interact, they suggest that the three protein components might interact and form a trimer. Considering the similar phenotypes of *EF1*_*pro*_:*amiR-*Mp*EFL*^Mp*MIR160*^ and Mp*lux*^*ge*^ lines, and overlapping expression domains, it is likely that these three proteins form a complex also in *M*. *polymorpha*.

### Overexpression of Mp*LUX* inhibits cell elongation and alters differentiation in the meristematic region

**Fig 7:**
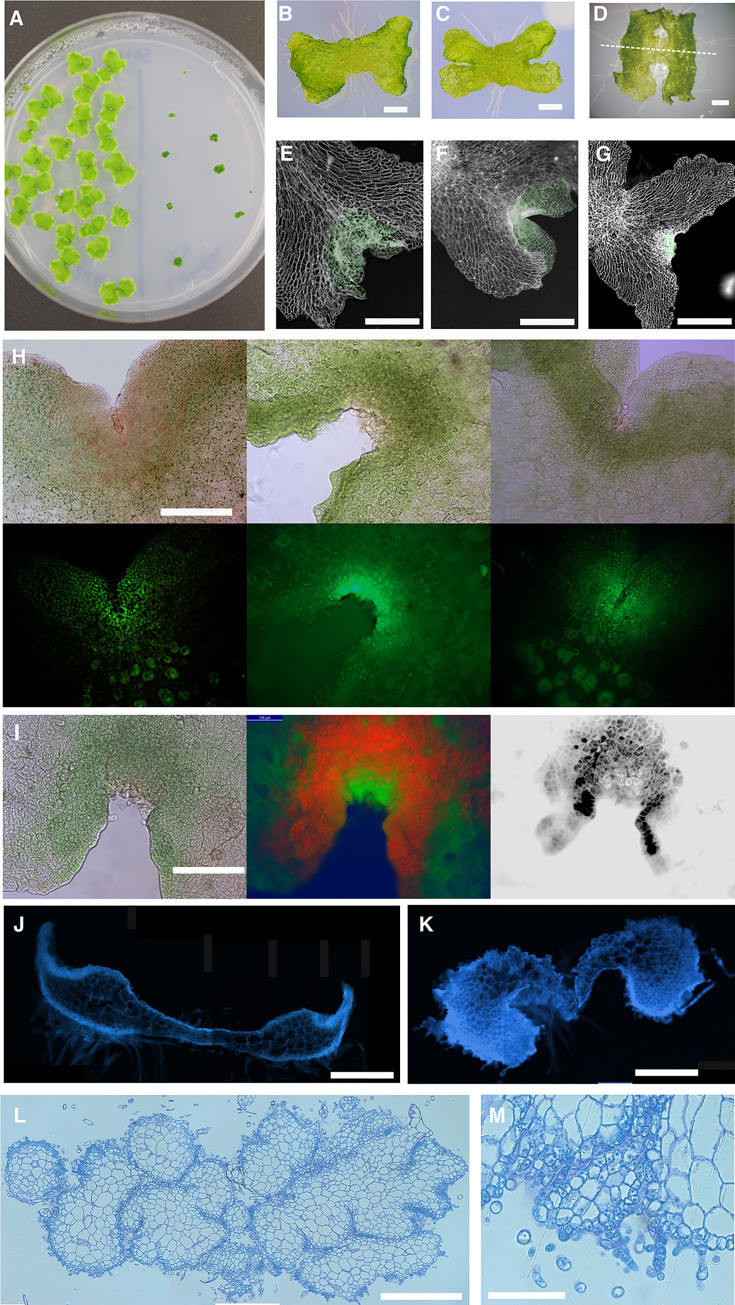
Overexpression of Mp*LUX* inhibits cell elongation and differentiation. (A) 3-week-old wild-type (Upp5, left) and *EF1*_*pro*_:Mp*LUX#1* (right) gemmalings grown on standard growth medium. (B-D) *EF1*_*pro*_:Mp*LUX-GR#1* gemmalings. (B) Grown on standard growth medium for one week. (C) Grown on medium supplemented with 25 μM dex for one week. (D) Grown on medium supplemented with 25 μM dex for two weeks. The dashed line is drawn through two imaginary points where the apical notches would be in the wild type, and illustrates how vertical longitudinal sections were cut in (J) and (K). (E) 3-day-old *EF1*_*pro*_:*amiR-*Mp*LUX* ^Mp*MIR160*^ gemmaling on dex medium. (F) 3-day-old wild-type gemmaling on dex medium. (G) *EF1*_*pro*_:Mp*LUX-GR* gemmaling on dex medium. Thallus tissue derived from cell division at the apical notches after gemma germination are indicated in green. (H) 3-day-old dex-treated gemmalings imaged in bright field (top) and fluorescent light (bottom) to reveal EdU stained S-phase nuclei. From left to right, *EF1*_*pro*_:*amiR-*Mp*LUX*^Mp*MIR160*^, wild type, *EF1*_*pro*_:Mp*LUX-GR*. (I) 3-day-old *EF1*_*pro*_:Mp*LUX-GR* gemmaling grown on 25 μM dex. The plant was imaged in bright field (left), excitation to reveal green fluorescence from EdU stained nuclei and red chlorophyll autofluorescence (middle), and UV light to reveal fluorescence from SR2200 stained cell walls (right). (J) Fluorescence image of vertical longitudinal section of 10-day-old wild-type gemmaling grown on 25 μM dex, stained with SR2200 to reveal cell walls. (K) Fluorescence image of vertical longitudinal section of 10-day-old *EF1*_*pro*_:Mp*LUX-GR* gemmaling on dex stained with SR2200 to reveal cell walls. (L-M) Vertical transverse sections of 3-week-old *EF1*_*pro*_:Mp*LUX-GR* gemmaling in 10x (L), and 40x (M) magnification. Bars are 500 μm in (A,B,C,D,E,F,G,J,K,L) and 100 μm in (H,I,M).

From the loss-of-function phenotype we hypothesized that overexpression of Mp*LUX* would lead to dwarfed plants. To test this hypothesis we produced several independent lines harboring *EF1*_*pro*_:Mp*LUX* or *EF1*_*pro*_:Mp*LUX-GR* constructs, generating constitutive ectopic expression of Mp*LUX* or the MpLUX protein fused to the glucocorticoid receptor (GR), allowing dex-mediated import of MpLUX-GR to the nucleus, respectively. Without dex, *EF1*_*pro*_:Mp*LUX-GR* lines grew and developed as the wild type, but with dex they became dwarfed and identical to *EF1*_*pro*_:Mp*LUX* lines (Fig 7A–7D). Previous studies have not found any effect by dex on wild type *M*. *polymorpha* growth or development [36, 43, 46]. We also overexpressed Mp*EFL* and Mp*ELF3* as fusions to GR, creating several dex-inducible *EF1*_*pro*_:Mp*ELF3-GR* and *EF1*_*pro*_:Mp*EFL-GR* lines (S7 Fig). All Mp*ELF3* and Mp*EFL* gain-of-function lines were phenotypically identical to the wild type when grown in standard growth conditions, and they did not respond at the phenotypic level to dex. To test if Mp*LUX* could be rate-limiting for an EC we analyzed RNAseq data from wild type (Tak-1) grown in LL, and sampled at twelve time points over two days [35]. We found that the reads per kilobase and million mapped reads (RPKM) for Tak-1 (average under LL) was 16.6 for Mp*LUX*, 33.4 for Mp*ELF3* and 40.1 for Mp*EFL*. The average mRNA levels of Mp*EFL* and Mp*ELF3* were thus at least twice as high as those of Mp*LUX*. The level of MpLUX could thus be rate-limiting for the function of the EC in *M*. *polymorpha*.

The *EF1*_*pro*_:Mp*LUX* gain-of-function lines all developed as small, compact, dark green balls, suggesting cells did not elongate and differentiate as in the wild type (Fig 7A). An identical phenotype was seen when gemmae from *EF1*_*pro*_:Mp*LUX-GR* plants were placed on medium containing dex (Fig 7B–7D). To better understand the observed phenotype, we studied the growth of young gemmalings of *EF1*_*pro*_:*amiR-*Mp*LUX* ^Mp*MIR160*^, wild-type and *EF1*_*pro*_:Mp*LUX-GR* plants grown on dex-containing medium (Fig 7E–7G). Initially, growth of gemmalings was mainly based on cell expansion of cells already present in the mature gemma. In this phase, gemmae from all genotypes expanded similarly in LL, but the rate of expansion was largest in the *EF1*_*pro*_:*amiR-*Mp*LUX* ^Mp*MIR160*^ genotype (S1 Video). After about two days, cell division at the two apical notches became notable in wild type and *EF1*_*pro*_:*amiR-*Mp*LUX* ^Mp*MIR160*^, and the expansion of those cells started to contribute to the thallus sheet area. At this stage the development of *EF1*_*pro*_:Mp*LUX-GR* started to deviate markedly from that of the other two genotypes when grown on dex (Fig 7E–7G). In the *EF1*_*pro*_:Mp*LUX-GR* gemmae, no new sheet developed from the apical notch area. Instead a dense area of cells developed, suggesting cell division without subsequent cell expansion. We therefore explicitly examined cell division through EdU staining of S-phase cells in the apical notch of wild type, *EF1*_*pro*_:*amiR-*Mp*LUX* ^Mp*MIR160*^ and *EF1*_*pro*_:Mp*LUX-GR* lines. The assays showed that cell division was high in all three lines (Fig 7H and 7I). Thus, the lack of an expanding sheet derived from the apical notch of *EF1*_*pro*_:Mp*LUX-GR* was not due to a lack of cell division but a lack of cell expansion and/or correct differentiation of the dividing cells. Tissues resulting from intense cell division showed strong autofluorescence from chloroplasts suggesting differentiation into chlorenchyma-like cells (Fig 7I). The resulting massive accumulation of dense cells at the apical region was visible after staining cell walls of *EF1*_*pro*_:Mp*LUX-GR* (Fig 7I–7K). Contrary to the regulated patterning of the tissues in the thallus, as observed in the wild type (Fig 5A and 5B; S5 Fig) [47], continued growth of *EF1*_*pro*_:Mp*LUX-GR* on dex-supplemented medium resulted in a massive accumulation of dense non-expanding cells, surrounding a core of larger vacuolated cells, that eventually made up most of the growing structure as observed in transverse sections of dex-treated *EF1*_*pro*_:Mp*LUX-GR* gemmalings (Fig 7L and 7M). In these sections, no single growth point (apical cell region) was found. Instead it appears the cell mass expands at several independent sites around the structure, forming lobes. These data suggest that MpLUX may promote cell proliferation or alternatively attenuate proper cell differentiation.

## Discussion

Previous studies have shown that Mp*LUX* and Mp*EFL* have a role in the *M*. *polymorpha* circadian clock [32, 35]. In the present study we show that MpLUX, likely as part of an evening complex with MpELF3 and MpEFL, has a significant effect also on growth. Mutants lacking Mp*LUX* function, or with reduced Mp*EFL* function, showed increased and epinastic growth of thalli, a light green phenotype resulting from fewer photosynthesizing chlorenchyma cells, and fewer and smaller gemma cups with germinating gemmae. More detailed analyses of growth revealed that both more and larger cells accompanied the larger thallus size of Mpl*ux*^*ge*^ lines. The thallus was also thinner, not only due to fewer chlorenchyma cells, but also due to more flattened parenchyma cells. In contrast, ectopic expression of Mp*LUX* promoted cell proliferation and suppression of correct organ differentiation.

It is possible that Mp*LUX* has a function to attenuate differentiation of cells to enable further meristematic activity within air chambers and chlorenchyma cells to produce enough photosynthetic filaments to fill up those chambers. Such lack of MpLUX activity could explain the reduced number of chlorenchyma cells and thus the thin and light green phenotype of Mp*lux*^*ge*^, as well as the formation of callus like phenotypes from undifferentiated cells, or alternatively cells with chlorenchyma-like identity, after ectopic expression of the gene.

The first stage of air chamber development includes the formation of intercellular apertures through a schizogenous process at a distance of four to five cells from the apical cell [48]. The further growth of the air chamber depends on coordinated anticlinal cell division of the roof and floor cells of the developing air chamber. However, the divisions of the protodermal roof cells ceases, while the cells of the floor retain full meristematic activity. In some of them a new axis of growth is established, i.e. the cells grow into the chamber and become polarized. This cell divides further to form a filament of three to five cells. Branching occurs occasionally through oblique divisions [48]. Thus, meristematic activity in the sub-protodermal layer and filament cells is vital for proper development of photosynthetic filaments. As one of Mp*LUX* main expression domain is within air chambers and chlorenchyma cells, it is conceivable that lack of Mp*LUX* activity in Mp*lux*^*ge*^ results in reduced meristematic activity in floor and filament cells leading to fewer filament cells.

This proposed function of Mp*LUX* is analogous to the one suggested for another MYB transcription factor in *M*. *polymorpha GEMMA CUP-ASSOCIATED MYB1* (*GCAM1*) [46]. Knockout mutants of *GCAM1* fail to develop gemma cups, where the gene has a predominant expression domain, whereas ectopic expression of the gene results in proliferation of undifferentiated cells [46]. The authors suggested that *GCAM1* might maintain undifferentiated cells of the floor of gemmae cups to enable to formation of gemma initials. Mp*gcam1* mutants did not produce any gemma cup under assayed growth conditions, whereas Mp*lux*^*ge*^ seemed to produce a similar number of air chambers as wild type. Thus, the main obstacle in air chamber development in Mp*lux*^*ge*^ seems to be the formation of the rows of chlorenchyma cells constituting the photosynthetic filaments from the floor cells. Mp*LUX* is also strongly expressed in developing gemmae as well as the floor of gemma cups. Similarly to mutants of GCAM1, Mp*lux*^*ge*^ also lacked gemma cups under normal growth conditions. Thus, MpLUX might have a role also in the promotion of gemma production.

In *M*. *polymorpha*, endogenously or exogenously increased levels of auxin results in an epinastic growth pattern, and distortions in the shape of thallus e.g., protrusion of air chambers and gemma cups [37, 42, 43]. The shape distortions may partly be due to increased cell elongation in the dorsal epidermal layer [43]. Consistent with the increased auxin levels in Mp*lux*^*ge*^ gemmalings [35], the mutant thalli showed epinastic growth and bulging of the epidermal layer. The Mp*lux*^*ge*^ thalli also grow larger as observed when applying low doses of exogenous auxin [49]. The similarities between the phenotypes of Mpl*ux*^*ge*^ and those of other genotypes with increased auxin levels suggest that the increased auxin levels in Mpl*ux*^*ge*^ might cause an enlarged epidermal layer and ectopic thallus-like outgrowth in the midrib region, which in turn could explain at least part of the increased thallus size seen in Mpl*ux*^*ge*^.

LUX and the EC in Arabidopsis also has a dual role, functioning within the circadian clock, as well as downstream of the clock in the control of growth [50]. However, the mechanisms revealed so far by which *LUX* in Arabidopsis and Mp*LUX* control growth are not conserved. The pathway identified by which LUX affect growth includes transcriptional repression of *PIFs* that in turn promote elongation growth. Surprisingly, the present and other studies [36, 38] could not identify a role for Mp*PIF* in promoting growth. We did not observe any effect on Mp*PIF* expression in Mp*lux*^*ge*^ knockout lines, and detailed analysis of growth rate in Mp*pif*^*ko*^ mutants rather suggested a weak attenuating function of Mp*PIF* on growth. It is possible that this contrasting growth-related role of PIFs in liverworts and angiosperms is coupled to evolution of the strong thermomorphogenic and photomorphogenic responses typical of angiosperms [51]. In angiosperms, both shade-avoidance signaling and thermomorphogenesis signaling promote elongation growth and PIFs constitute a central hub in this signaling in concert with phytochromes and the EC [15]. Thus, the incorporation of PIFs in these signaling pathways might have been a key event in the evolution of these responses.

## Material and methods

### Plant growth and cultivation

*Marchantia polymorpha* ssp. *ruderalis* Swedish accessions Uppsala (Upp) 1, Upp5 and Upp14, Australian male and female [42], Takaragaike (Tak) -1 and Tak-2, as well as transgenic lines, were grown aseptically on agar solidified Gamborg’s B5 medium [52] (PhytoTechnology Laboratories, Lenexa, KS, USA), pH 5.5. Plants were grown under cool white fluorescent light (50–60 μmol photons m^-2^ s^-1^) in 16:8 h, light:dark cycles (long days, LD) at 20°C or as otherwise stated in the text.

Tobacco (*Nicotiana benthamiana*) was grown in a glass house facility in LD and 60% humidity, with 22°C day and 18°C night temperatures.

To induce GR-fusion proteins we grew gemmalings on standard growth medium supplemented with 25μM dexamethasone (dex) dissolved in ethanol.

### Construction of plasmids

All primers are listed in S1 Table. All PCR fragments were cloned into pENTR/D-TOPO (Thermo Fisher, Uppsala, Sweden) and sequenced before transfer to binary destination vectors or yeast expression vectors.

A 1.7 kb Mp*EFL*_*pro*_ fragment was amplified using primers ME643+ME644. Mp*EFL*_*pro*_:*LUC* was created by LR-cloning Mp*EFL*_*pro*_ into pMpGWB431 [29]. A 5.5 kb Mp*LUX*_*pro*_ fragment was previously amplified and cloned [26]. Mp*LUX*_*pro*_:*GUS* was created by LR-cloning Mp*LUX*_*pro*_ into pMpGWB104 [29]. The Mp*PRR*_*pro*_:*LUC* plasmid is described in Linde et al. [26].

The full length coding domain sequences of Mp*LUX*, Mp*ELF3* and Mp*EFL* were PCR amplified using primers ME385+ME386, ME381+ME382 and ME383+ME384, respectively. Mp*ELF3* was sub-cloned into the NotI site of pFL61 [53], while Mp*LUX* and Mp*EFL* were sub-cloned into the NdeI/SalI sites of pGBKT7 and the XmaI/SalI sites of pGAD-GH (BD Biosciences, Clontech Laboratories, Mountain View, CA, USA), respectively. *EF1*_*pro*_:Mp*LUX* was created by moving the Mp*LUX* full length CDS into pMpGWB103 [29].

Coding domain sequences without stop codons of Mp*LUX*, Mp*ELF3* and Mp*EFL* were PCR amplified using primers ME385+CPEP86, ME381+CPEP88 and ME383+CPEP87, respectively. n/cLUC fusions in binary plasmids were created by LR-cloning the obtained CDSes into plasmids pDEST(GFP)-GW N-LUC and pDEST(FP611)-GW C-LUC [45].

For inducible constructs *EF1*_*pro*_:Mp*LUX-GR*, *EF1*_*pro*_:Mp*EFL-GR* and *EF1*_*pro*_:Mp*ELF3-GR* the CDSes without STOP-codon were transferred to pMpGWB113 by LR cloning [29].

### Plant transformation

Constructs were introduced into Agrobacterium, strain GV3101. *M*. *polymorpha* sporelings were transformed essentially as previously described [28]. Transformed sporelings were plated on selective media: Gamborg’s B5 with 10 μM Hygromycin and/or 10 μM G418, plus 200 μM Timentin (PhytoTechnology Laboratories, USA).

### Gene expression analysis

RNA extraction was performed with an RNeasy Plant Mini Kit (Qiagen), cDNA was synthesized using SuperScript III Reverse Transcriptase (Thermo Fisher) and analysed by qRT-PCR as previously described [26, 35]. Primers are listed in S1 Table. Mp*EF1α*, Mp*ACT* and Mp*APT3* were used for normalization [54].

For sampling of RNA we used four replicates–two biological replicates (plants of individual transgenic lines or individual wild type lines), each with two experimental replicates (pools of two or three individually grown plants of the same transgenic line or wild type line) [32]. We did one cDNA-synthesis reaction from each RNA sample. In all sampling we used large gemmalings harboring adult tissues, but with no visible gemma cups. We sampled plant material grown in constant light conditions (LL) at 12 time points over 44 hours. Plants were entrained in ND and sampling started after 12 hours in LL [32]. Test of statistically significant overall average expression differences between lines were performed with a linear model in R (aov) [55].

To estimate the relative expression of Mp*LUX*, Mp*ELF3* and Mp*EFL*, reads per kilobase and million mapped reads (RPKM) were estimated from an RNA-seq experiment interrogating circadian rhythm in *M*. *polymorpha* [35]. Averages over twelve time points over two days in continuous light sampled in triplicates every six hours were used.

Luciferase assays were performed as described by Linde et al. [26], using an ImagEM CCD camera (Hamamatsu Photonics). For GUS assays [56], plants were incubated in GUS solution (0.5 mM potassium ferrocyanide, 0.5 mM potassium ferricyanide, and 1 mM X-Gluc) at 37°C overnight. Subsequently, plants were cleared with ethanol and then stored in 10% glycerol solution.

### Protein interaction analyses

Yeast hybrid assays were performed using the BD Matchmaker system (BD Biosciences, Clontech Laboratories, Mountain View, CA, USA) using plasmids based on pGBKT7 and pGAD-GH, together with pFL61 [53]. *Saccaromyces cerevisiae* strain PJ69-2A, harboring the reporters *HIS3* and *ADE2* [57], was used essentially as previously described [58].

Agrobacterium infiltration and measurements of reconstituted LUC signals in floated whole leaves were performed essentially as described previously at 3 days after infiltration [26, 59], using an ImagEM CCD camera (Hamamatsu Photonics). To enhance the signal intensity we co-infiltrated the Tombusvirus P19 RNA silencing suppressor [60–62]. The binary plasmid backbones expressed the fluorescent proteins EGFP and eqFP611 to verify successful infiltration of the nLUC- and cLUC-containing plasmids, respectively [45]. Agrobacterium strain GV3101 was used for these experiments.

### Measurements of thallus surface area and growth rates

Gemmae from wild type (Upp5), *EF1*_*pro*_:*amiR-*Mp*LUX*^Mp*MIR160*^ and *EF1*_*pro*_:*amiR-*Mp*EFL*^Mp*MIR160*^ were grown in neutral day photoperiod (12:12 h, light:dark cycles; ND) on aseptically on agar solidified Gamborg’s B5 medium for four days and transferred to constant light supplemented with infra-red light (IR). Images were captured every hour with an IR-sensitive camera. Images were imported to ImageJ as image stack and converted to binary images for calculation of area. For analysis of Mp*pif*^*ko*^ mutants in ND and LL, one Mp*pif*^*ko*^ line and one restored line, behaving as wild type (Mp*PIF*_*pro*_:Mp*PIF* Mp*pif*^*ko*^; [38], were grown for four days and transferred to ND supplemented with IR light. Imaging was performed over four days in ND followed by another four days in LL. Image stacks were analyzed as described above. For analysis of Mp*pif*^*ko*^ in short day photoperiod (8:16 h, light:dark cycles; SD), two independent mutant lines and two independent restored lines were grown, imaged and analyzed as above, but in SD (eight days) instead of ND (four days) plus LL (four days). It should be noted that *M*. *polymorpha* is not growing in constant darkness [35]. Statistical analysis of growth rates was conducted by testing for differences in slope (the interaction term genotype*time (G*T) in linear regression) using the aov package in R. The square root of area was used as dependent variable.

### EdU staining

To visualize S-phase cells, staining was performed using the Click-iT™ EdU Cell Proliferation Kit for Imaging, Alexa Fluor™ 488 dye (Life Technologies, Eugene, OR, USA), largely following the manufacturers protocol. Gemmae were grown in 12-well plates on 0.25x Gamborg’s B5 medium with 0.25% agar. 100 μl of 20 μM EdU was added after 48 or 76 hours. After 24 hours of additional growth, gemmae were fixed by adding 500 μL 3.7% formaldehyde in PBS. After washing twice with 500 μL 3% BSA in PBS, incubation with 500 μL 0.5% Triton-X in PBS for 30 min, and washing with 500 μL 3% BSA in PBS, gemmae were incubated for 30 min in 250 μL reaction cocktail. After additional washing in 3% BSA in PBS, gemmae were mounted under coverslips on taped slides.

### Sectioning and microscopy

Wild type and Mp*lux*^*ge*^*-9* were grown for three weeks in LD and then fixed in FAA (10% formaldehyde, 5% acetic acid, 50% ethanol). Mp*LUX*_*pro*_:*GUS* lines were grown for three weeks and then GUS stained as described above, before being fixed in FAA. *EF1*_*pro*_:Mp*LUX-GR* was grown for three weeks on plates supplemented with dex, as described above. After fixation the material was dehydrated in an ethanol series.

To produce sections of Mp*lux*^*ge*^*-9*, *EF1*_*pro*_:Mp*LUX-GR*, and wild type thalli, tissue was first incubated in a 1:1 solution of 99.5% ethanol:infiltration solution (50 ml historesin base and 0.5 g activator powder from Leica historesin embedding kit #7022 31731 supplemented with 1 ml PEG400) for 3 hours in room temp whereafter tissue was transferred to concentrated infiltration solution for further incubation in fridge overnight. Embedding in a 15:1 mix of infiltration solution and hardener was carried out at room temperature in plastic Histomolds of 6 x 8 mm (Leica). 6 μm thick transversal sections obtained using a Microm HM 355S microtome with a glass knife were transferred to water-covered microscopy slides kept on a 42°C heating table. Once the water evaporated the dried-in sections were stained in 0.01% Toluidine blue (w/v, 0.1M phosphate buffer pH: 7.0) for 2 min and washed three times in water for 1 min. Sections of GUS-stained Mp*LUX*_*pro*_:*GUS* reporter tissue was produced in the same way but here sections were made 10 μm thick and Toluidine blue staining was omitted. Images of both Toluidine blue-stained Mp*lux*^*ge*^*-9*/wild type sections and GUS-stained Mp*LUX*_*pro*_:*GUS* sections were obtained using an Axioscope A1 microscope, an AxioCam ICc 5 camera, and the Zen Blue software (Zeiss), and Adobe PHOTOSHOP CC was used to adjust intensity and contrast in images.

### Measurements of sectioned material

Cell number, cell width, air chamber size, circularity and aspect ratio were measured from transverse sections such as the one shown in S5 Fig, using the free software Inkscape (https://inkscape.org) and ImageJ [63]. Individual cell and tissue measurements were traced and outlined in scalable vector graphics format. Further, 16-bit threshold binary modifications were produced to separate and calculate the outline, size and shape of respective cell, from equations described previously (https://imagej.nih.gov/ij/docs/menus/analyze.html).

## Supporting information

S1 Fig Number of chlorenchyma cells in each measured air chamber in the wild type and the Mp*lux*^*ge*^*-9* mutant, plotted against air chamber size. Chlorenchyma filament cells were counted in 38 and 60 air chambers from six Mp*lux*^*ge*^*-9* sections and ten wild type sections, respectively. The boxplot in Fig 3C is based on these data.
(TIF)

S2 Fig There is no size difference between the air chambers measured to estimate the number of chlorenchyma cells per area unit. Graphs show the average air chamber area in 38 and 60 air chambers of Mp*lux*^*ge*^*-9* and wild type, respectively. Error bars show SD. Two-tailed t-test, *P* = 0.26. This graph is more clearly illuminating what is shown on the Y-axis in S1 Fig.
(TIF)

S3 Fig Micrographs of transverse sections of GUS-stained 3-week-old thalli from Mp*LUX*_*pro*_:*GUS#2*,*7*,*9*. A) Overview of signal in Mp*LUX*_*pro*_:*GUS#2*, sectioned through two apical notches. (B) Section just behind the apical cell in thallus of Mp*LUX*_*pro*_:*GUS#7*. (C,D) Air chambers, chlorenchyma and parenchyma cells in Mp*LUX*_*pro*_:*GUS#9* (C) and Mp*LUX*_*pro*_:*GUS#2* (D). (E) Young gemma cup with gemmae of various sizes and developmental stages in Mp*LUX*_*pro*_:*GUS#2*.
(TIF)

S4 Fig Each dorsal epidermal cell in transverse sections of wild type and the Mp*lux*^*ge*^*-9* mutant, from midrib to thallus tip, plotted against individual cell width. This figure shows the data the boxplots in Fig 3E, 3F are based on. Cell measurements were done in the ten and six sections of wild type and Mp*lux*^*ge*^*-9*, respectively, that were also used in Fig 3C and S1 Fig.
(TIF)

S5 Fig Transverse section of a wild type thallus. Lines were added for counting cell number and size at five positions along the thallus: 0, 25, 50, 75 and 100% of the total length from midrib to tip of the thallus margin. A box was added to indicate part of parenchyma used for circularity and aspect ratio measurements.
(TIF)

S6 Fig EGFP and eqFP611 signals in all tobacco leaves infiltrated with split-LUC plasmids verifies successful infiltration. Panels are displayed in the same order as in Fig 6. Left panels show signals after using a GFP filter. Right panels show signals after using an RFP filter. Only one of the two leaves from each panel shown in Fig 6 is displayed here.
(TIF)

S7 Fig Expression of GR-fusions for Mp*ELF3* and Mp*EFL* in transgenic lines. Graph shows *GR* expression levels in samples of two biological replicates of wild type and four independent lines each of *EF1*_*pro*_:Mp*ELF3-GR* and *EF1*_*pro*_:Mp*EFL-GR*. The average of WT1 and WT2 was set to 1. Because the wild type has no *GR* fusion gene, the signal in WT1 and WT2 is only noise.
(TIF)

S1 Table Oligonucleotides used in this study. (PDF)

S1 Video Growth rates of wild type, *EF1*_*pro*_:*amiR-*Mp*LUX*^MpMIR160^ and *EF1*_*pro*_:Mp*LUX-GR* on dexamethasone. Gemmae of separate genotypes are placed in triplicates in rows: top row, *EF1*_*pro*_:*amiR-*Mp*LUX*^Mp*MIR160*^; middle row, *EF1*_*pro*_:Mp*LUX-GR*; bottom row, wild type. The plate was supplemented with 25μM dex.
(AVI)
