# Supplementary figures and images for: PIF-independent regulation of growth by an evening complex in the liverwort Marchantia polymorpha
Source: PLoS One. 2022 Jun 16;17(6):e0269984. doi: 10.1371/journal.pone.0269984 (PMC9202859; doi:10.1371/journal.pone.0269984)

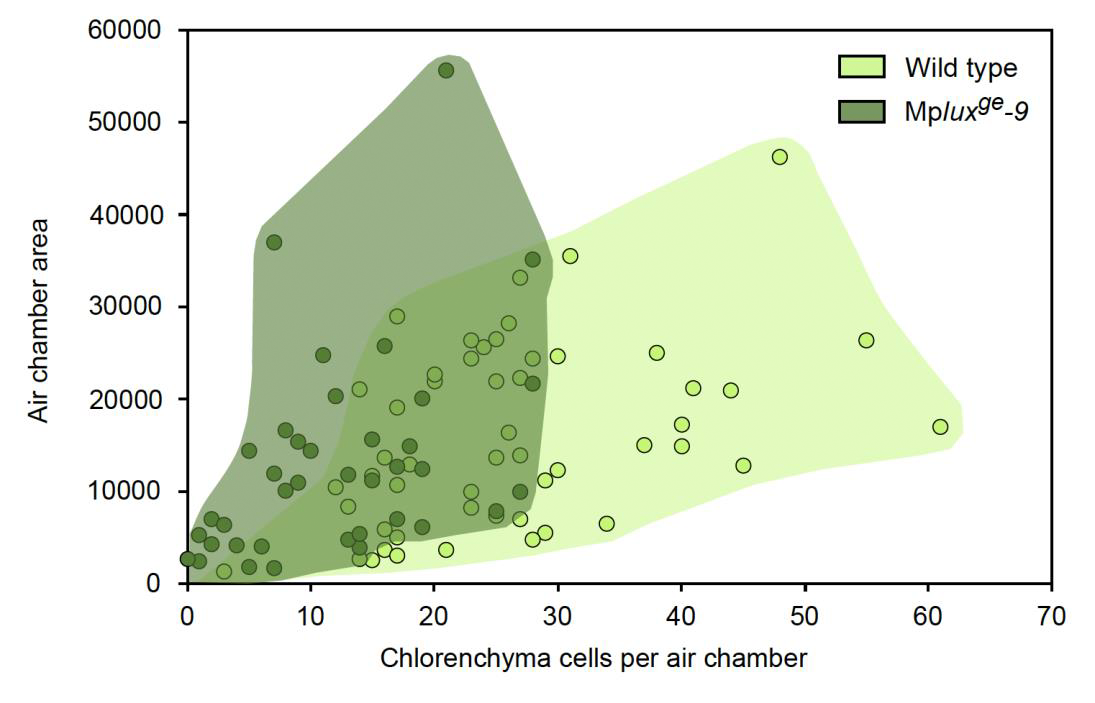

Supplement: S1 Fig — Chlorenchyma filament cells were counted in 38 and 60 air chambers from six Mpluxge-9 sections and ten wild type sections, respectively. The boxplot in Fig 3C is based on these data. (TIF) [file pone.0269984.s001.tif]

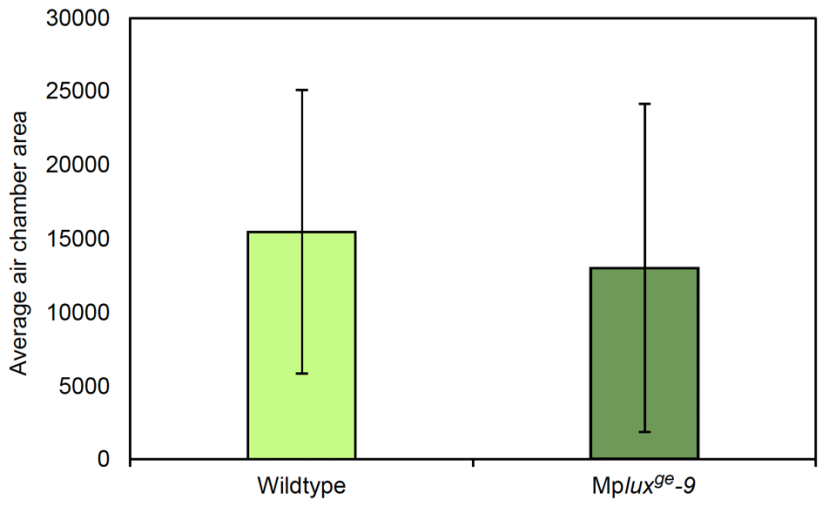

Supplement: S2 Fig — Graphs show the average air chamber area in 38 and 60 air chambers of Mpluxge-9 and wild type, respectively. Error bars show SD. Two-tailed t-test, P = 0.26. This graph is more clearly illuminating what is shown on the Y-axis in S1 Fig. (TIF) [file pone.0269984.s002.tif]

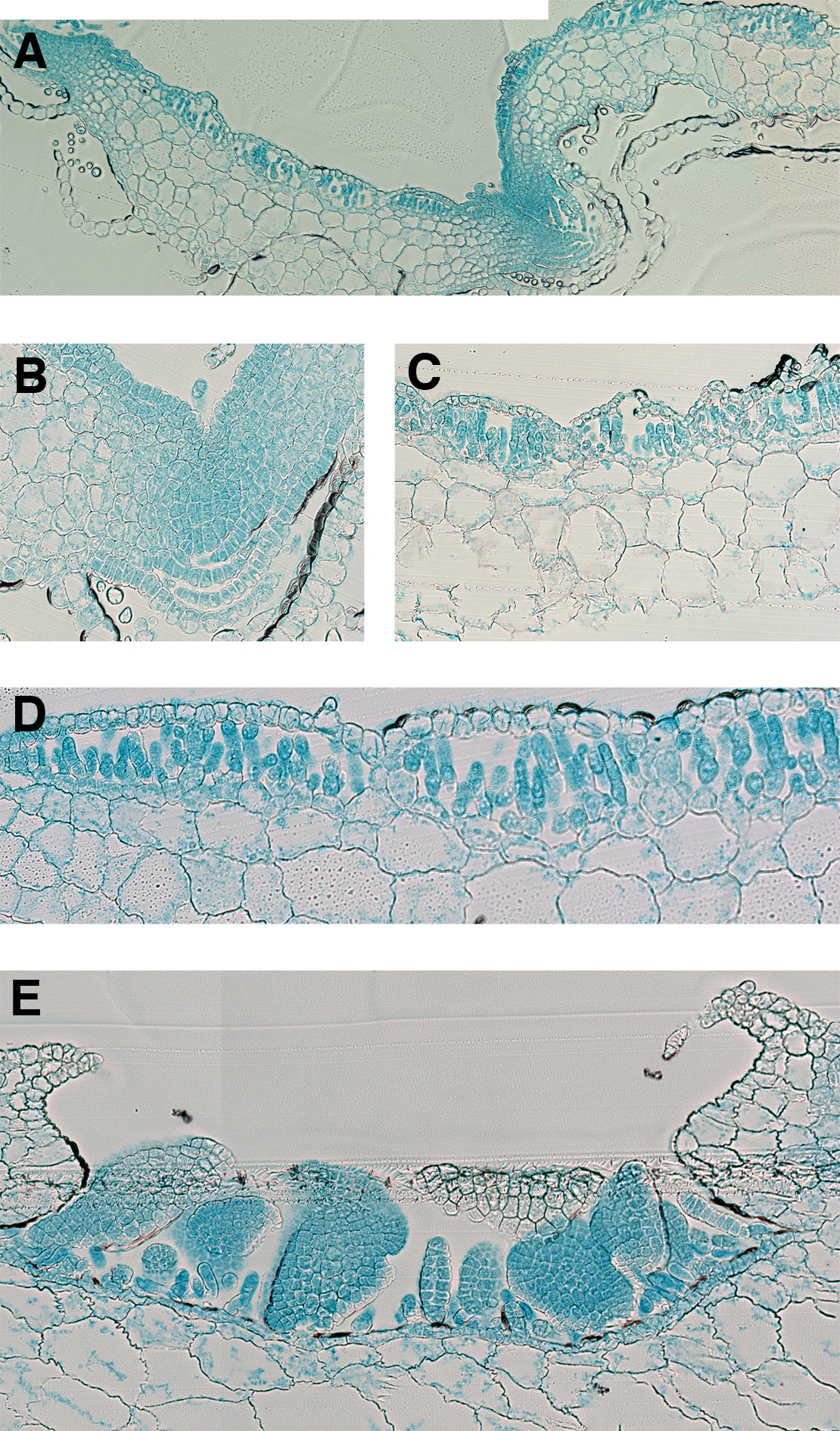

Supplement: S3 Fig — A) Overview of signal in MpLUXpro:GUS#2, sectioned through two apical notches. (B) Section just behind the apical cell in thallus of MpLUXpro:GUS#7. (C,D) Air chambers, chlorenchyma and parenchyma cells in MpLUXpro:GUS#9 (C) and MpLUXpro:GUS#2 (D). (E) Young gemma cup with gemmae of various sizes and developmental stages in MpLUXpro:GUS#2. (TIF) [file pone.0269984.s003.tif]

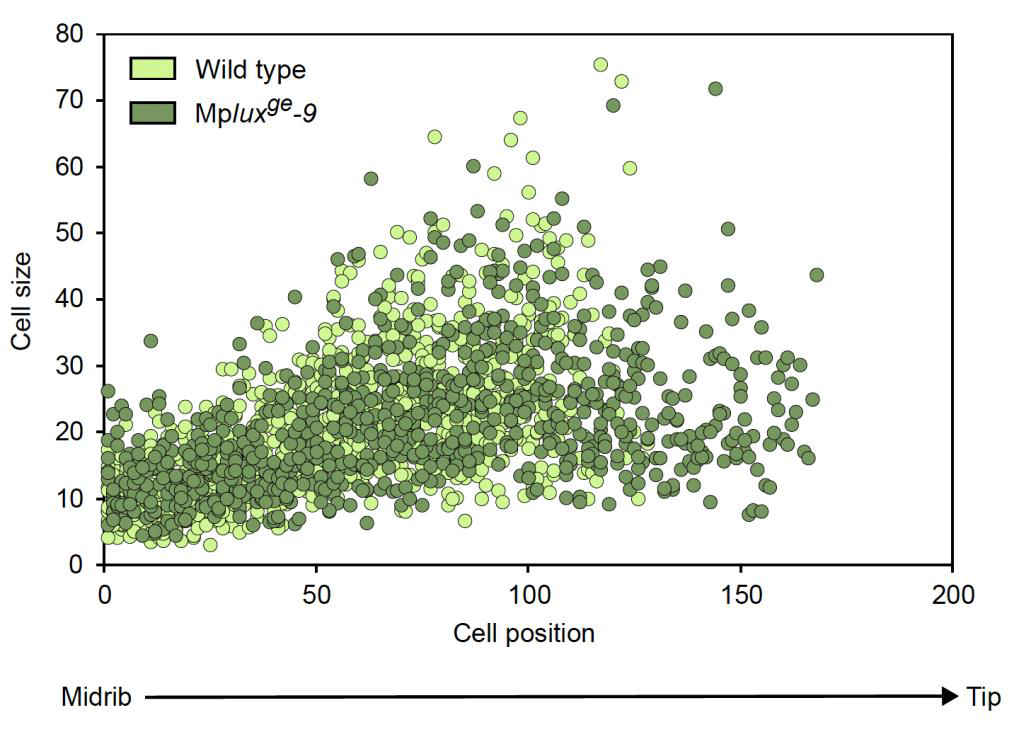

Supplement: S4 Fig — This figure shows the data the boxplots in Fig 3E, 3F are based on. Cell measurements were done in the ten and six sections of wild type and Mpluxge-9, respectively, that were also used in Fig 3C and S1 Fig. (TIF) [file pone.0269984.s004.tif]

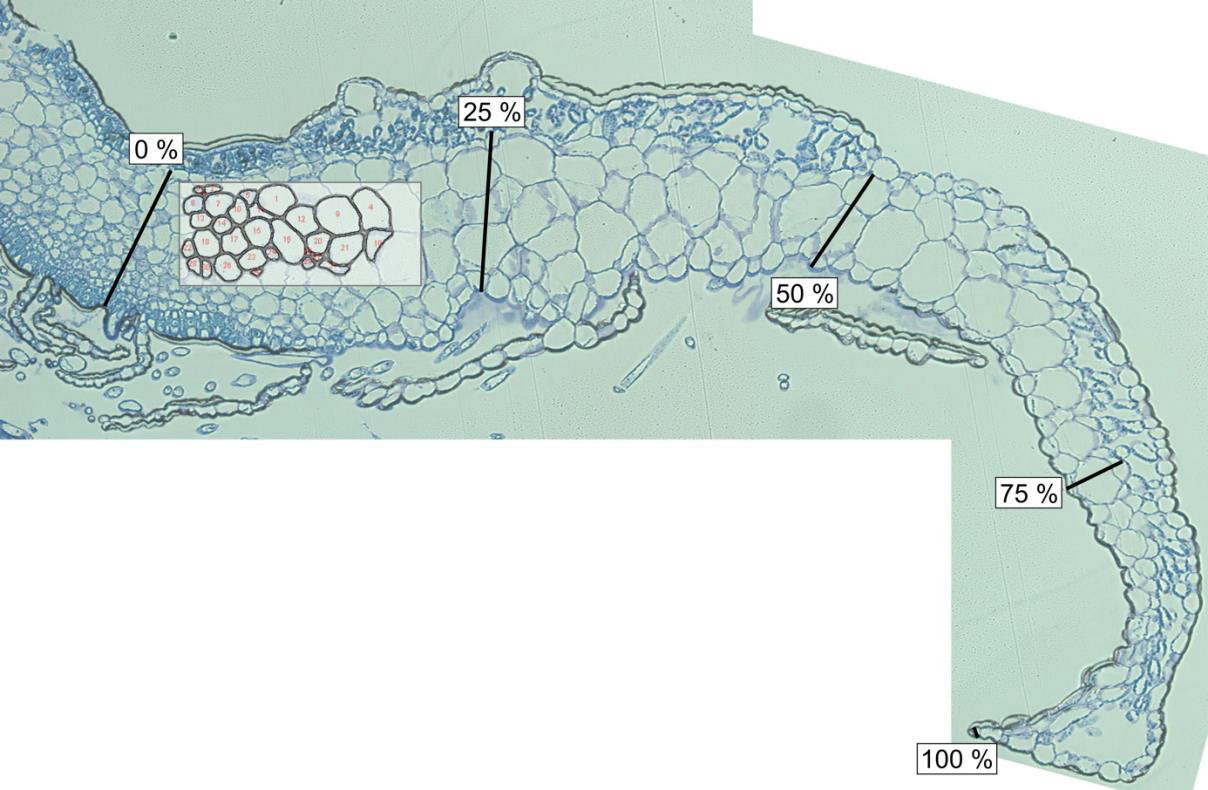

Supplement: S5 Fig — Lines were added for counting cell number and size at five positions along the thallus: 0, 25, 50, 75 and 100% of the total length from midrib to tip of the thallus margin. A box was added to indicate part of parenchyma used for circularity and aspect ratio measurements. (TIF) [file pone.0269984.s005.tif]

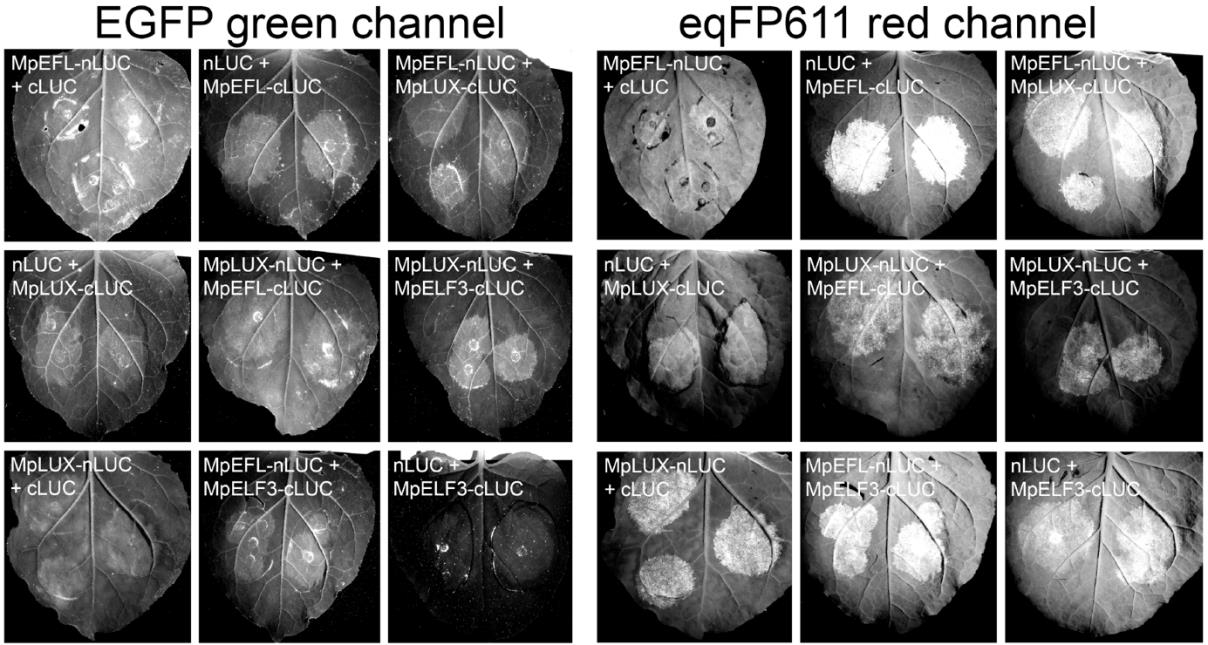

Supplement: S6 Fig — Panels are displayed in the same order as in Fig 6. Left panels show signals after using a GFP filter. Right panels show signals after using an RFP filter. Only one of the two leaves from each panel shown in Fig 6 is displayed here. (TIF) [file pone.0269984.s006.tif]

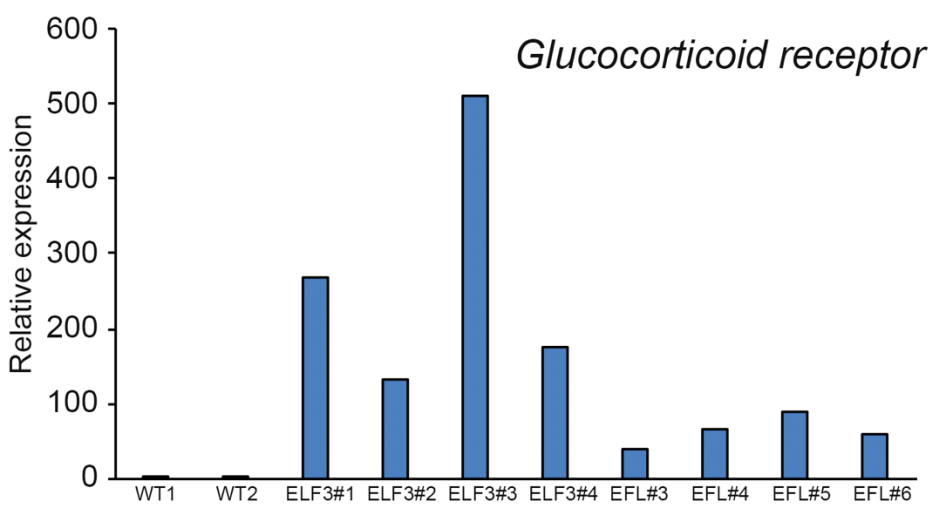

Supplement: S7 Fig — Graph shows GR expression levels in samples of two biological replicates of wild type and four independent lines each of EF1pro:MpELF3-GR and EF1pro:MpEFL-GR. The average of WT1 and WT2 was set to 1. Because the wild type has no GR fusion gene, the signal in WT1 and WT2 is only noise. (TIF) [file pone.0269984.s007.tif]
