# Supplementary material for: PIF-independent regulation of growth by an evening complex in the liverwort Marchantia polymorpha
Source: PLoS One. 2022 Jun 16;17(6):e0269984. doi: 10.1371/journal.pone.0269984 (PMC9202859; doi:10.1371/journal.pone.0269984)
Supplement: S1 Table — (PDF) [file pone.0269984.s008.pdf]

S1 Table. Oligonucleotides used in this study.

Primer name	Sequence - 5' to 3'	Comments
CPEP86	GTTGCAAACTGCAAGCCATTTCTC	Rev MpLUX CDS no stop
CPEP87	GTCCATAGATGACCGTACATTCTTCA	Rev MpEFL CDS no stop
CPEP88	CTGCTGACGCTGCTTCTGAATAGA	Rev MpELF3 CDS no stop
ME367	CGAAAGCCCAAGAAGCTACC	Fwd MpAPT qRT-PCR[1]
ME368	GTACCCCCGGTTGCAATAAG	Rev MpAPT qRT-PCR[1]
ME369	AGGCATCTGGTATCCACGAG	Fwd MpACT qRT-PCR[1]
ME370	ACATGGTCGTTCCTCCAGAC	Rev MpACT qRT-PCR[1]
ME381	caccgcggccgcATGATgGGCACGaAgGAT	Fwd MpELF3 CDS
ME382	gcggccgcTTACTGCTGACGCTGCTT	Rev MpELF3 CDS
ME383	cacccccgggaATGGACACCGATGCCT	Fwd MpEFL CDS
ME384	gtcgacTTAGTCCATAGATGACCGT	Rev MpEFL CDS
ME385	cacccatatgATGGCAGCGATGAAGGA	Fwd MpLUX CDS
ME386	tgtcgacCTAGGTTGCAAACTGCAAG	Rev MpLUX CDS
ME402	CTTGGTTGACTTTGGGCAAT	Fwd MpYUC2 qRT-PCR[2]
ME403	CCGACCTTGTCTTTCAGCTC	Rev MpYUC2 qRT-PCR[2]
ME444	ACCCGAGGTGTTGTATGCAG	Fwd GR qRT-PCR
ME445	TCACTTGACGCCCACCTAAC	Rev GR qRT-PCR
ME643	caccTTCTTAACCCTAGGAAAAGCATGT	Fwd MpEFL promoter
ME644	GACTCGCAAATCTCTACTGGAAG	Rev MpEFL promoter
ME649	CCGCAATCCCAACACTTACT	Fwd MpPIN1 qRT-PCR
ME650	AGCGTCCGAGAGAATGCTAA	Rev MpPIN1 qRT-PCR
ME655	GGATATACGGCGTCTTTGGA	Fwd MpABCB3 qRT-PCR
ME656	CCGAAGTCCGGGTTGTAGTA	Rev MpABCB3 qRT-PCR
ME665	CCGAGATCCTGACCAAGG	Frw MpEF1 qRT-PCR[1]
ME666	GAGGTGGGTACTCAGCGAAG	Rev MpEF1 qRT-PCR[1]
ME699	AGCTTGAGCAGTTGGACGAT	Fwd MpGH3A qRT-PCR
ME700	GTTTCTCCCTCTCCCTCCAC	Rev MpGH3A qRT-PCR
ME727	TTCTGCGAATGGCTAAAACC	Fwd MpWIP qRT-PCR
ME728	ATTGTCCTCGGTGGTGAATG	Rev MpWIP qRT-PCR
ME744	GGACCAAGTGATTCGCTCTC	Fwd MpTAA qRT-PCR[2]
ME745	ACAATGCAGCCTGGAAGAGT	Rev MpTAA qRT-PCR[2]
ME758	AGGTTCCGAAGCTCAACAGA	Fwd MpPIF qRT-PCR
ME759	ATCCACGGAGGAATTGACAG	Rev MpPIF qRT-PCR

Notes

1. Saint-Marcoux D, Proust H, Dolan L, Langdale JA. 2015. Identification of reference genes for real-time quantitative PCR experiments in the liverwort *Marchantia polymorpha*. *PLOS ONE* 10: e0118678.
2. Lagercrantz U, Billhardt A, Rousku SN, Ljung K, Eklund DM. 2020. Nyctinastic thallus movement in the liverwort Marchantia polymorpha is regulated by a circadian clock. *Scientific Reports* 10: 1–9.
